# It’s not a virus! Reconceptualizing and de-pathologizing music performance anxiety

**DOI:** 10.3389/fpsyg.2023.1194873

**Published:** 2023-11-10

**Authors:** Rebecca Herman, Terry Clark

**Affiliations:** ^1^Centre for Performance Science, Royal College of Music, London, United Kingdom; ^2^Mount Royal Conservatory, Calgary, AB, Canada

**Keywords:** music performance anxiety, MPA, musicians, interventions, theoretical literature review

## Abstract

Music Performance Anxiety (MPA) is one of the most widespread and debilitating challenges facing musicians, affecting significant numbers of performers in terms of both their personal and professional functioning. Although numerous interventions exist to target MPA, its prevalence remains unchanged since the first large-scale studies of the 1980s, indicating that available interventions are having limited impact. This review synthesizes and critiques existing literature in order to investigate possible reasons for the limited efficacy of current approaches to managing MPA. Key concepts discussed include conceptual and methodological challenges surrounding defining MPA, theoretical perspectives on MPA’s etiology and manifestation, and the coping strategies and interventions used to manage MPA. MPA has predominantly been investigated pathologically and defined as a negative construct manifesting in unwanted symptoms. Based on this conceptualization, interventions largely seek to manage MPA through ameliorating symptoms. This review discusses possible reasons why this approach has broadly not proved successful, including the issue of relaxation being both unrealistic and counterproductive for peak performance, issues associated with intentionally changing one’s state creating resistance thus exacerbating anxiety, and focusing on the presence of, rather than response to, symptoms. Despite 50 years of research, MPA remains an unsolved enigma and continues to adversely impact musicians both on and off the stage. Reconceptualizing MPA as a normal and adaptive response to the pressures of performance may offer a new perspective on it, in terms of its definition, assessment and management, with practical as well as theoretical implications.

## 1. Introduction

Public perception associates musical performance with entertainment, pleasure and relaxation (Hildebrandt et al., 2012). Indeed, the stage can be a place of creative freedom and artistic excellence, eliciting incredibly positive, even euphoric, states (Kenny, 2011). However, although musicians report higher levels of job satisfaction than other occupational groups (Harper, 2002), significant numbers experience occupation-related physical and psychological health problems that interfere with their personal and/or professional functioning, one of the most prevalent and debilitating of which is music performance anxiety (Fernholz et al., 2019).

Music Performance Anxiety (MPA) occurs in response to perceived threat and manifests physiologically, cognitively, emotionally, and behaviorally, ranging in intensity from normal stress intrinsic to performance to extreme levels of terror (Steptoe, 2001; Burin and Osório, 2017). MPA does not occur in isolation but interacts with stressors associated with a highly demanding, competitive and insecure work environment (Vervainioti and Alexopoulos, 2015) as well as numerous personality traits, including perfectionism, trait anxiety, cognitive style and coping style (Burin et al., 2019).

Experiencing acute anxiety can be psychologically distressing; over time, chronic anxiety can affect all aspects of performers’ lives, including wellbeing, identity, self-worth and relationships (Kenny, 2011; Burin and Osório, 2017; Matei et al., 2018). *In extremis*, MPA may lead to post-traumatic stress disorders (Moura and Serra, 2021). Research indicates that prolonged neuroendocrine activation and overstimulation of the sympathetic nervous system can codetermine, or even cause, diseases including heart attacks and strokes (Benson and Klipper, 2009; Gomez et al., 2018). Alongside threatening performers’ health and quality of life, MPA can reduce performance enjoyment, cause performance avoidance and impair performance quality (Kenny, 2011). At its most severe, MPA can constitute an occupational disability, terminating studies and careers (Orejudo et al., 2017; Fernholz et al., 2019). Sternbach (1993) describes the toxicity of MPA as making life preceding an important performance an *“unremitting purgatory”* for both performers, and those around them.

Given MPA’s potentially devastating effects on musicians’ lives and/or careers (Nagel, 2010), it is unsurprisingly one of the most widely researched topics in the fields of performance science and performing arts medicine, and there is now a substantial body of scholarship investigating MPA across a wide range of populations from a diverse range of theoretical and methodological perspectives. You may be wondering why on earth we need *YET ANOTHER* theoretical review of MPA. Despite decades of research and the development of numerous interventions, MPA’s prevalence remains unchanged since the first large-scale studies in the 1980s (Fishbein et al., 1988; Fernholz et al., 2019), indicating that available interventions are not having a meaningful impact. MPA continues to pose a significant occupational challenge to musicians of all ages, genres, and nationalities, suggesting that gaps remain in our understanding of it (Fernholz et al., 2019). This theoretical review aims to explore possible reasons why MPA remains an unsolved enigma. Sections 2–6 will synthesize and critique existing research across the key concepts and issues in MPA research: Prevalence (2), Definitions (3), Etiology (4), Manifestation (5) and Management (6). Section 7 (Discussion) will then summarize current conceptualizations of MPA, before suggesting a paradigmatic reconceptualization.

## 2. Prevalence

MPA is described as ubiquitous (Nagel, 2010), an epidemic (McGrath, 2012) and universal (Brandfonbrener, 1999). But just how widespread really is MPA? Studies investigating prevalence among student and professional musicians report figures between 24 and 96%, indicating that MPA affects significant, yet ambiguous, numbers of musicians. While it is clear that MPA poses a serious challenge to students and professionals alike, the variability of findings casts little light on the issue of prevalence, precluding a clear understanding of the scale of the issue. This ambiguity can be attributed to methodological limitations within studies and heterogeneity across studies (Brugués, 2011a), as summarized in Table 1.

**Table 1:** MPA prevalence among conservatoire students and professional musicians including key methodological characteristics of the studies.

Study	Sample	Response rate	Definition used	Measures used	Key findings: MPA prevalence
Fishbein et al. (1988)	*N* = 2,212 musicians in 48 American orchestras	55%	Stage fright defined as the most extreme form of MPA (no operational definition of either construct).	Questionnaire developed by authors and ICSOM Music Medicine Committee. Participants circled relevant medical problems and indicated severity, defined by perceived impact on performance. Questionnaire not validated.	Stage fright was the most frequently reported serious physical/psychological problem, with 24% finding it problematic and 16% severely problematic.
Steptoe (1989)	*N* = 65 orchestral musicians (UK)	87%	MPA, performance anxiety, and stage fright used interchangeably; (no operational definition of any construct).	State–Trait Anxiety Inventory (State scale) (STAI-S) (Spielberger, 1983)	32% of participants were highly performance anxious.
Wesner et al. (1990)	*N* = 302 University of Iowa music students/faculty	66.5%	Anxiety	Questionnaire developed by the authors measuring *“distress due to performance anxiety, impairment caused by anxiety, and treatment sought for it”* on a Likert scale (1–5) (p. 178)	21% reported “marked distress” and 40% experienced “moderate distress” while performing due to anxiety. 16.5% reported impaired performance quality due to anxiety. 16.1% believed performance anxiety had adversely impacted their careers.
Cox and Kenardy (1993)	*N* = 32 music students (UK)	53.3%	Performance anxiety is not defined.	State–Trait Anxiety Inventory (Trait scale) (STAI-T) (Spielberger, 1983) Performance Anxiety Questionnaire (PAQ) (Cox and Kenardy, 1993) (unvalidated) Social Phobia & Anxiety Inventory (SPAI) (Turner et al., 1989)	100% of participants reported experiencing anxiety in performance; 84% considered anxiety to impair their performance; 96% reported experiencing performance-related stress.
Bartel and Thompson (1994)	*N* = 204 musicians in Canadian orchestras	21%	Questionnaire asks about ‘stress related to performance’ (not defined).	Questionnaire developed by the authors based on a focus group of musicians from the Toronto Symphony Orchestra as well as reviewed literature.	The authors concluded that professional orchestral life is ‘extremely stressful’, with a higher percentage of musicians experiencing stress than in the general workforce. The most effective coping strategy reported was beta-blockers.
Van Kemenade et al. (1995)	*N* = 155 musicians in Dutch orchestras	23.8%	Performance anxiety is not defined.	Questionnaire developed by the authors (unvalidated).	59% had experienced performance anxiety severe enough to impact their professional and/or personal lives, with anxiety manifesting up to months prior to important performances.
James (2000)	*N* = 1,639 musicians from 57 orchestras (worldwide)	50%	Anxiety sufficient to have an adverse impact on playing.	Questionnaire developed by the author (not included with publication).	70% of respondents reported anxiety severe enough to affect their performance, with 16% experiencing this degree of anxiety at least weekly.
Kaspersen and Götestam (2002)	*N* = 126 conservatoire students in Norway	96.2%	Anxiety is not defined.	Questionnaire developed by the authors based on reviewed literature and knowledge of the Norwegian conservatoire system.	36.5% experienced MPA severely enough to require help. Only 8% reported no anxiety during musical performance.
Studer et al. (2011)	*N* = 190 music students in Swiss universities.	22%	*‘Trac’,* translates as ‘stage fright’. (Questionnaire in French)	Based on lack of universally agreed upon definition, MPA was assessed *via*: State Trait Anxiety Inventory (State scale) (STAI-S) (Spielberger, 1983) to measure MPA’s emotional dimension. Rating of the degree to which participants considered stage fright to be a problem (0–4 scale)	One third of participants considered stage fright to be problematic. 20% of participants felt their performances were often impaired by stage fright.
Gembris and Heye (2012)	*N* = 2,536 musicians in German orchestras	26%	*‘Lampenfieber’* translates loosely as stage fright. Not operationally defined here.	Questionnaire (in German) developed by authors based on qualitative pilot study (*n* = 12) and literature.	Over 90% suffered from MPA, with 50% reporting experiencing MPA to a small degree, 30% moderately and 13% severely.
Zakaria et al. (2013)	*N* = 55 undergraduate music students	92%	Uses terms ‘performance anxiety’ and ‘stage fright’ interchangeably. Defined as *“a constant, continuously distressful and impairment of performance skills in public context which may affect individual’s musical aptitude training and level of performance”* (p. 227)	Questionnaire developed by the authors incorporating (1) demographics, (2) timeframe of performance anxiety, (3) symptoms and (4) coping strategies.	96% of participants reported experiencing performance anxiety sometimes during performance; 88% believed that their anxiety adversely impacted their playing.
Topoğlu et al. (2018)	*N* = 220 musicians in Turkish orchestras	57%	Uses Kenny (2009) definition.	Turkish version of the K-MPAI (Kenny, 2009)	81.8% experienced MPA, with 60% describing MPA as impairing their performances

Most problematic are issues surrounding definitions and measurement tools (see Table 1). Unsurprisingly, prevalence varies enormously depending on the definition criteria used (Osborne and Kirsner, 2022). There is no consensus regarding what actually constitutes MPA, both in terms of conceptual clarity *within*, and heterogenous terminology *across*, studies. The diversity of terms used, as well as the very different constructs they represent, makes it impossible to know how many musicians experience the same phenomenon. This ambiguity is reflected in the diverse range of measures deployed. Within studies, the lack of a clear operational definition makes it impossible to know what exactly is being measured; across studies, the heterogenous assessment tools used preclude comparison between studies, making it impossible to build a strong evidence base (Kenny et al., 2014; Gembris et al., 2018).

MPA poses a major occupational challenge to significant numbers of musicians at all stages of professional development. However, if prevalence ranges from 24% to ubiquitous, it seems evident we are not all talking about the same phenomenon. Why does this matter? We suggest that the divergence regarding how many musicians experience MPA points to a more fundamental issue – how can we help musicians to manage a thing without knowing what that *thing* is? A phenomenon which affects a quarter of a particular population has very different implications than a phenomenon which affects *all* of a population. Arguably, the lack of a universal definition of MPA precludes comprehensive understanding, and thus effective management, of MPA. We now therefore turn to the challenge of defining MPA.

## 3. Definitions and theoretical perspectives

“*Performance anxiety, like pornography, is easily recognised but difficult to define.”*

This quote by Lederman (1999, p. 117) illustrates a fundamental issue in MPA research – musicians and researchers alike know, intuitively, what MPA is, yet the phenomenon still lacks a common definition (Fernholz et al., 2019). This section will discuss the key challenges surrounding defining MPA: the heterogeneity of terms used to label the phenomenon, and the diverse conceptual constructs these terms represent.

### 3.1. Terminology

Across literature, different terms are used interchangeably and with conflicting hierarchies of severity (Studer et al., 2011). Pre-2009, authors largely created their own label and definition, applying only to their own study, precluding comparability of data and the formation of a coherent MPA theory. Some authors consider stage fright the most extreme manifestation of performance anxiety (Fishbein et al., 1988; Steptoe, 1989; Brodsky, 1996; Senyshyn, 1999), whereas others place stage fright below performance anxiety on their continuum of severity (Fehm and Schmidt, 2006). Van Kemenade et al. (1995) investigate performance anxiety that affects personal and/or professional functioning and (James, 1998) measures only anxiety which adversely impacts performance, which is both vague and problematic. That stage fright has been considered both more AND less severe than MPA by different researchers adds further confusion to the prevalence statistics reported in Table 1.

Since 2009, researchers have increasingly adopted the definition offered by Kenny (2009) (see Section 3.3). However, discrepancies remain across the literature (Osborne and Kirsner, 2022), inhibiting understanding of the construct being investigated and posing a real challenge to the field in terms of methodological rigor, diagnostic criteria, evaluating prevalence and severity and designing appropriate interventions. In addition to heterogenous terminology, there is divergence regarding the nature of the phenomenon these terms are labeling. We now turn to various conceptualizations of MPA: negative, positive, both and intrinsic to performance. For clarity, this review will only use the term ‘MPA’.

### 3.2. Conceptualizations of MPA

#### 3.2.1. MPA as negative

MPA is largely researched through a pathological lens (Pecen et al., 2016) and described using medicalized language: *‘condition’* (Barros et al., 2022; Moreno-Gutierréz et al., 2023), *‘syndrome’* (Lederman, 1999), *‘disabling’* (Brantigan et al., 1979; Lederman, 1999), *‘illness’* (Harper, 2002), *‘disease’* (Sousa et al., 2016; Fernholz et al., 2019), and *‘disorder’* (Kenny, 2011; Fernholz et al., 2019; Mumm et al., 2020), requiring *‘treatment’* (Spahn, 2015). The International Classification of Diseases defines performance anxiety as a specific phobia (ICD-10; Dilling and Freyberger, 2015), and the Diagnostic and Statistical Manual for Psychiatric Disorders (DSM 5; Falkai et al., 2015) codes it as a performance-only subtype of social anxiety disorder (SAD) (both cited in Mumm et al., 2020, p. 76–77).

Although some studies indicate correlations between MPA and aspects of SAD (Clark and Agras, 1991; Osborne and Kenny, 2005), recent evidence suggests that MPA and SAD are conceptually and empirically unrelated (Wiedemann et al., 2021). It appears that although MPA and SAD can manifest comorbidly and share similar aspects, they are distinct constructs (Bögels et al., 2010) and SAD is arguably not a useful diagnostic classification (Ruggiero, 2012), even in more extreme manifestations of MPA. In addition to a lack of empirical support for a connection between SAD and MPA, there are conceptual issues with linking the two constructs.

Although fear of evaluation is common to both MPA and the social anxiety disorders MPA tends to be diagnostically clustered with (Hays, 2009), there are arguably qualitative differences between performing and other social situations in terms of the physical and psychological challenges involved in performance (Kenny, 2011; Chang-Arana et al., 2018) as illustrated by the following quote:

*“It is one thing to avoid cocktail parties (or funerals) because you feel uncomfortable in the presence of others. It is quite another, however, to diagnose a tenor as having a social phobia as a result of the following: He functions quite well in most social situations but feels at least momentarily daunted anticipating the thousand pairs of eyes and ears watching and listening as he opens his mouth to sing "Comfort Ye, My People," the initial vocal moment in Handel's Messiah”* (Hays, 2009 p. 105).

Conflating the two situations risks pathologizing an entirely normal response to the well documented demands of performance (Antonini Philippe et al., 2022) and the very real risks of negative evaluation in musical studies and careers: When dependent on the positive opinion of audition panels, colleagues or audiences to pay one’s mortgage, fear of evaluation arguably seems not so unreasonable!

#### 3.2.2. MPA as positive

An alternative approach posits that MPA is facilitative and enhances performance by generating extra vitality, excitement, alertness, focus and spontaneity (Hamann and Sobaje, 1983; Nagel, 1993; Roland, 1994; Kantor-Martynuska et al., 2018) (See Gannon (2019) for discussion of how MPA can facilitate performance). This quote by virtuoso Andalusian guitarist Pepe Romero illustrates how experiences commonly associated with anxiety can be experienced positively:

*“The feeling you get backstage…butterflies in your stomach…your knees shake so much that you can hardly stand, and…you feel like you’re about to throw up?? I LOVE that!! It’s because we’re like a rechargeable battery: those sensations tell us that energy is flowing into our bodies, energy that we need to give to the music and to the audience. So, I don’t fight those feelings, I let them flow right into me, and when I step on stage, I let them flow out to the public so that they become energised too!”* (Cited in Helding, 2016, p. 89).

This account only mentions physiological activation, omitting the psychological dimension commonly associated with MPA descriptions (Kenny, 2011). The interaction between performers’ physiological and psychological experiences, as well as performers’ interpretations of physiological activation seem central to our understanding of MPA, and will be discussed in Section 5. As research has largely focused on debilitating MPA, those who experience MPA positively and/or manage it successfully are underrepresented in scientific literature, highlighting an important gap in current understanding.

#### 3.2.3. MPA as both negative and positive

There have been three key attempts to clarify the debate between MPA as wholly negative or positive. Firstly, conceptualizing MPA as a singular but multi-faceted construct encompassing both adaptive and maladaptive components (Wolfe, 1989). Secondly, viewing MPA as two separate constructs (facilitative and debilitative), although there remains disagreement regarding whether the constructs are distinct (Mor et al., 1995) or exist along a linear continuum (Spahn et al., 2016; Brugués, 2019). Thirdly, delineating MPA (negative) from an entirely different, positive, construct (*‘performance energy’*/*‘boost’*) describing the performance-enhancing state of anticipation which generates excitement, energy and positive tension (Langendörfer et al., 2006; Nagel, 2010; Simoens et al., 2015). Using one label (‘MPA’) to define two negatively correlated constructs seems bound to confuse. Indeed, it is difficult to think of any other psychological construct where the same word is used to describe two opposite phenomena. Further research is required to untangle these issues.

#### 3.2.4. MPA as intrinsic to performance

Some researchers shift the focus entirely from performer to performance: Sternbach (1993) refers not to MPA, but instead to the performer’s capacity to cope with the anxiety of performing, implying that performing is inherently stressful and the issue is how individuals manage that stress, a view supported by numerous subsequent researchers including Sataloff et al. (1999), Hays (2009) and Spahn (2015) (Stressors associated with musical performance will be discussed in Section 4.2.3). Brodsky (1996) reconceptualized MPA as pertaining to the nature of a career in music in terms of performance and occupational stress, rather than to personality deficiencies or underlying psychopathology. He offered the alternative label of ‘Music-Performers’ Stress Syndrome’ (M-PSS), including the option of various specifiers (e.g., level of intensity) to reflect the individuality of performers’ experiences of MPA. While this seems a valuable contribution to the field, it was never adopted by other researchers. Brodsky additionally proposed a linear continuum of severity moving from ‘career stress’ at one end, to ‘tension in performance’ to ‘performance anxiety’ and finally to ‘stage fright’ at the other end (see Brodsky, 1996, p. 91). While an interesting perspective, Brodsky does not explain why performers would experience performance stress so dramatically differently, or what delineates the four stages. If stress is intrinsic to performance, it remains unclear what constitutes ‘normal’ stress and when stress becomes MPA.

#### 3.2.5. Alternative conceptualizations

There have been a few notable recent attempts to offer a completely different perspective on MPA, including Lawrence (2019), who reconceptualizes MPA as performers’ unconscious desires manifesting in psychological or somatic form, and Skoogh and Frisk (2019) who investigate MPA from an artistic, rather than psychological, perspective. They theorize that MPA is not an individual problem, but rather a structural issue relating to complex interactions between perfectionism and performance values found in western classical music. While offering real insight, neither has yet impacted the predominantly pathologizing approach to defining MPA.

### 3.3. Kenny’s definition

The definition most widely used today is offered by Kenny (2010):

*“Music performance anxiety is the experience of marked and persistent anxious apprehension related to musical performance that has arisen through specific anxiety-conditioning experiences. It is manifested through combinations of affective, cognitive, somatic and behavioural symptoms and may occur in a range of performance settings, but is usually more severe in settings involving high ego investment and evaluative threat. It may be focal (i.e., focused only on music performance), or occur comorbidly with other anxiety disorders, in particular social phobia. It affects musicians across the lifespan and is at least partially independent of years of training, practice, and level of musical accomplishment. It may or may not impair the quality of the musical performance” (p. 433)*.

Although this definition is used by most contemporary MPA studies, there are issues worth discussing. Firstly, defining MPA by its “*symptoms*” perpetuates the pathologizing narrative, conjuring up images of illness and disease. As well as the philosophical issues with medicalizing MPA (see Section 7), if it can be facilitative for some, or simply inherent to performance, then the *presence* of “symptoms” may not be the key issue to understanding or managing MPA.

Secondly, MPA is “*at least partially independent of years of training, practice, and level of musical accomplishment*.” Given that MPA is reported by individuals of all levels of training, experience and expertise (including celebrated artists such as Chopin, Casals, Rubinstein, Horowitz, and Rachmaninoff), MPA can presumably be *entirely*, not *partially*, independent of expertise (Brugués, 2011a; Kantor-Martynuska et al., 2018).

Lastly, the strand “*MPA may or may not impair the quality of the musical performance”* tells us very little about the complex relationship between MPA and performance quality, which will be discussed in Section 5.3. It also omits the impact MPA can have on the *experience* of performing, regardless of whether quality is affected. Across the extensive landscape of MPA literature, a recurring theme is the significant variability with which MPA can manifest, ranging from performance-enhancing, to minimally negative, to debilitating, to career-ending and varying in terms of regularity, performance-setting and manifestation (Nagel et al., 1989; Van Kemenade et al., 1995; Miller and Chesky, 2004; Fehm and Schmidt, 2006; Patson and Loughlan, 2014; Lawrence, 2019). This complexity and multidimensionality is arguably not yet reflected in the prevailing approach to defining MPA.

In sum, the field still lacks a cohesive MPA definition, and we suggest that the main obstacles are (1) terminological and (2) conceptual ambiguities. Regarding terminology – using one term (‘MPA’) to describe a phenomenon which can be positive, mildly challenging, debilitating or a completely normal part of a performer’s life, seems at best unhelpful, at worst deeply problematic. Without a shared language, it becomes impossible to ensure we are all talking about the same thing, which may partially explain the lack of success in supporting musicians to manage MPA – how can we manage a thing effectively without really knowing what that *thing* is? We urgently need a universal MPA definition to be able to effectively support performers experiencing it.

Regarding conceptual ambiguities, although many argue that the majority (if not all) of performers experience apprehension and nerves, and many experience nerves as positive and performance-enhancing (Powell, 2004), efforts to conceptualize MPA as beneficial or intrinsic to performance have been drowned out by the predominantly pathologizing approach (Lawrence, 2019), with significant implications for its perception, experience and management, as will be discussed in Sections 6.2.2 and 7.

Perhaps the issue is not whether MPA is definitively negative, positive or both, but that musicians clearly experience MPA in vastly different ways. The questions then become, what mediate these differences? How can we conceptualize MPA in a way that adequately captures its individuality, complexity and variability and leads to the development of interventions which can support musicians in real and tangible ways? We now turn to MPA’s etiology and manifestation in the hopes of addressing these fundamental issues.

## 4. Etiology

### 4.1. Theoretical perspectives on MPA’s etiology

There is consensus among researchers that MPA is a complex, multidimensional phenomenon produced by the interaction between numerous factors including genetics, biology, environmental stimuli, aversive conditioning experiences, conscious and unconscious anxiety triggers, psychological characteristics and factors relating to musical tasks and performance contexts (Papageorgi et al., 2007; Burin and Osório, 2017; Brugués, 2019). More specifically, there remains debate over which theory best accounts for MPA’s etiology. Theories posited (summarized in Table 2) include cognitive theories, behavioral theories, cognitive behavioral theories, physiological theories, psychoanalytic theory and Barlow’s Anxiety Model (2000), adapted to MPA by Kenny et al. (2004), Spahn (2015), Altenmüller and loannou (2016), Brugués (2019).

**Table 2:** Summary of key psychological theories explaining MPA’s etiology.

Cognitive theories	Cognitive theories emphasize the centrality of dysfunctional cognitions in the development and maintenance of MPA and their impact on physiology, emotions and behavior (Spahn, 2015).
Behavioral theories	Behavioral models conceptualize MPA as a classically conditioned fear attributed to traumatic learning experiences during performers’ formative years which lead to the development of maladaptive cognitions and behaviors (Clark, 1989; Spahn, 2015; Altenmüller and Ioannou, 2016). Different responses to similar learning experiences can be attributed to genetic/factors (Kenny, 2011).
Cognitive behavioral theories	Cognitive behavioral psychologists attribute MPA to negative cognitions and self-beliefs that undermine preparation and self-confidence (Altenmüller and Ioannou, 2016; Brugués, 2019), particularly catastrophizing and attention binding (Beck and Clark, 1988). The distinction between cognitive theories and cognitive behavioral theories of MPA remains unclear (Kenny, 2011).
Physiological theories	Physiological psychologists primarily attribute MPA to humans’ evolutionary stress response whereby perception of threat triggers a range of physiological reactions known as the fight/flight response. Modern threats include failure, humiliation, exposure and disappointing significant others/peers (Spahn, 2015; Altenmüller and Ioannou, 2016).
Psychoanalytic theory	According to psychoanalytic perspectives (including attachment theory), MPA symptoms arise from individual childhood experiences, including attachment with caregivers, and express conscious or unconscious internal conflicts (Kenny, 2011; Spahn, 2015). In this modality, the audience represents parental figures; fear of negative evaluation reflects feared loss of parental love. Unconscious fears manifest in negative cognitions and fears of losing control (Spahn, 2015). There remains minimal empirical support for this theoretical perspective (Brugués, 2019).
Barlow’s Anxiety Model (2000)	Kenny et al. (2004) adapted Barlow’s (2000) tripartite Anxiety Model to MPA, and this model has underpinned Kenny’s subsequent work on MPA. This adapted theory attributes MPA to the interaction between three dimensions of vulnerability: biological (personality traits), general psychological (early learning experiences) and specific psychological vulnerabilities (learned anxiety in response to specific environmental stimuli) (Barlow, 2000; Kenny, 2011; Gembris and Heye, 2012) Although the most comprehensive of these theories, the distinction between biological and learned traits seems somewhat arbitrary in terms of relevance for musicians seeking to manage MPA. While traits may be relatively stable, research shows their malleability, which problematizes classifying traits as biological.

While these various theoretical perspectives individually offer insight into specific dimensions of MPA’s etiology, none emerges as being comprehensive. Additionally, there is currently no widespread agreement among researchers to support any one theory, all of which lack empirical support (Goren, 2014; Fernholz et al., 2019). While these theories have all contributed significantly to current understanding of MPA (Kenny, 2011), there is a legitimate counterargument that generalizing about MPA’s etiology is futile, as its origins vary significantly from person to person (Brandfonbrener, 1990), arguably suggesting a utilitarian, rather than theoretical, approach.

One such utilitarian approach is Wilson’s (1994) framework, which divides known MPA risk factors into Person, Task, and Situation. Although this tripartite framework offers a pragmatic heuristic to conceptualize the multitudinous factors contributing to MPA’s etiology, two limitations are worth consideration. Firstly, it is clear from the extensive stress literature that it is the individual’s interpretation of a stimulus which causes stress, not the stimulus itself (Lazarus and Folkman, 1984). For example, not all musicians reporting similar occupational stressors experience them as equally stressful, or experience similar levels of MPA (Kenny et al., 2004), indicating that stimuli from the Task and Situation domains are subjective and thus filtered through *Person.* Secondly, the three categories are not distinct, but highly interconnected, with complex interactions both within and between domains. Indeed, one domain’s impact depends on the level of others (Valentine, 2002). Despite these limitations, this is arguably the best fit available, and has been adopted by subsequent researchers including Valentine (2002) and Burin and Osório (2017).

### 4.2. MPA risk factors

#### 4.2.1. Person

*Person* refers to intrapersonal factors which are associated with, and/or predict, MPA, and can therefore be considered risk factors. These include demographic variables (gender and sociocultural factors), biographical details (family, teachers and aversive conditioning experiences), personality traits, cognitive style and comorbid challenges. These factors (see Table 3) appear key to understanding how MPA develops and is sustained (Burin and Osório, 2017). Studies consistently report significant associations between MPA and high levels of maladaptive personality traits including maladaptive coping style (van Fenema et al., 2013; Thomas and Nettelbeck, 2014), low self-efficacy (Sinden, 1999; Liston et al., 2003; Kenny and Ackermann, 2015), maladaptive dimensions of perfectionism (Stoeber and Otto, 2006; Stoeber and Eismann, 2007; Kobori et al., 2011; Sarıkaya and Kurtaslan, 2018; Butković et al., 2022; Yang et al., 2022), low self-esteem (Papageorgi et al., 2007; Burin et al., 2019), negative affect (Zinn et al., 2000; Sadler and Miller, 2010), neuroticism (Langendörfer et al., 2006), susceptibility to anxiety (Stephenson and Quarrier, 2005), guilt/shame-proneness (Coşkun-Şentürk and Çırakoğlu, 2018) and trait anxiety (Osborne and Kenny, 2008; Burin et al., 2019).

**Table 3:** Intrapersonal factors associated with MPA.

Demographics	Gender	There is broad consensus that women of all ages experience MPA more widely and acutely than men (consistent with generalized anxiety) and that gender is a significant factor in predicting MPA (Papageorgi et al., 2007; Thomas and Nettelbeck, 2014; Burin and Osório, 2017; Fernholz et al., 2019).
	Sociocultural factors	James (2000) reported significant differences in the quantity of stress experienced from occupation-related stressors across different nationalities. No explanation was given to account for sociocultural differences. Based on his study investigating levels of self-esteem among American, Australian and Chinese music students, Brand (2004) concluded that self-perception and self-esteem differ across cultural groups. His findings indicate the importance of cultural differences in understanding the development, as well as the prevention and treatment, of MPA. Unfortunately, as no further studies have been conducted in this area (Brugués, 2019), understanding of this important topic remains minimal. This represents an important gap in understanding, as all *person* relationships to MPA are arguably culturally conditioned. Mumm et al. (2020) suggest that transcultural differences should be taken into account when diagnosing and treating MPA in musicians from different cultural backgrounds, such as individualistic versus collectivistic societies.
Biography factors	Family & Teachers	Influence of parental expectations (pressure), severity of pedagogical style and pressure from teachers (Brugués, 2019; Burin et al., 2019). See Kenny and Holmes (2015, 2018) and Wiedemann et al. (2020) for detailed discussion regarding the complex relationship between parenting and attachment style and MPA.
	Conditioning	Aversive experiences from past performances causing conscious or unconscious anxiety-triggers (Kenny, 2006; Osborne and Kenny, 2008; Burin et al., 2019). Negative experiences arguably increase the chance of perceiving performance situations as threatening, increasing the likelihood of MPA symptoms manifesting.
Personality traits	Coping style	Inadequate, or maladaptive, coping strategies significantly predict MPA (Sinden, 1999; van Fenema et al., 2013; Thomas and Nettelbeck, 2014); Difficulty coping with performance-related arousal and negative cognitions perceived as contributors to MPA (Burin et al., 2019).
	Self-efficacy	Several studies have shown a positive correlation between low self-efficacy and MPA (Sinden, 1999; Liston et al., 2003; Papageorgi et al., 2010) and a negative correlation between high self-efficacy and MPA (Craske and Craig, 1984). The significant association between self-efficacy and anxiety has been attributed to the interaction between low confidence in one’s ability to execute a task and physiological, psychological and behavioral anxiety symptoms (Bandura, 1991).
	Perfectionism	Previously thought to be a unidimensional construct, perfectionism is now defined as a multidimensional personality trait characterized by setting exceedingly high personal standards in search of flawless performance and tendencies toward overly critical self-evaluations (Hewitt and Flett, 1991). Perfectionism comprises both adaptive and maladaptive components – adaptive elements (perfectionistic strivings) are associated with positive behaviors, intrinsic motivation and achievements whereas maladaptive elements (perfectionistic concerns) are associated with negative behaviors and outcomes including excessive worry about making mistakes, self-doubt and negative responses to perceived failures or imperfections (Hewitt and Flett, 1991; Frost et al., 1993; Mor et al., 1995; Sinden, 1999; Stoeber and Otto, 2006; Stoeber and Eismann, 2007). Dimensions of maladaptive perfectionism are associated with depression, anxiety, excessive self-criticism, low self-esteem, disordered eating, maladaptive cognitions, performance dissatisfaction, fear of negative evaluation and burnout (Hewitt and Flett, 1991; McGrath, 2012; Zhukov, 2019). Studies of musicians reveal negative dimensions of perfectionism to strongly predict MPA as well as extrinsic motivation and psychological distress (Stoeber and Otto, 2006; Stoeber and Eismann, 2007; Kobori et al., 2011; Diaz, 2018). Pressure from self, associated with excessively high standards, is one of the most frequently cited causes of MPA (Kenny et al., 2014; Burin et al., 2019). The relationship between perfectionism and MPA is highly complex because both phenomena are multi-faceted and encompass both positive and negative dimensions, and because the relationship can be mediated by third variables, such as self-efficacy (Mor et al., 1995).
	Low self-esteem	Low self-esteem is associated with the prediction of MPA (Sinden, 1999; Papageorgi et al., 2007; Chan, 2011; Kenny et al., 2014; Burin et al., 2019). Musicians invest highly in their identity as performers and often struggle to disentangle their self-esteem from their musical competence – the perception that ‘failing’ in performance equates to failing as people increases performers’ vulnerability to anxiety (Sinden, 1999; Kenny, 2009).
	Negative affect	Studies have reported significant associations between negative affect and MPA (Zinn et al., 2000; Sadler and Miller, 2010), although the direction of this association is unclear.
	Neuroticism	Neuroticism and MPA are strongly associated (Valentine et al., 1995; Langendörfer et al., 2006; Thomas and Nettelbeck, 2014).
	Susceptibility to anxiety	Susceptibility to anxiety refers to an individual’s pattern of perceiving situations and anxiety symptoms as threatening or dangerous, and is a significant predictor of MPA, especially among women (Stephenson and Quarrier, 2005; Kenny, 2011; Burin and Osório, 2017).
	Trait anxiety	Anxiety is universally conceptualized as two-dimensional, comprising both state and trait elements – state anxiety is defined as a transient emotional state characterized by nervousness, apprehension, stress and heightened tension, whereas trait anxiety is a relatively stable personality characteristic whereby individuals tend to perceive situations as threatening (Spielberger, 2013; Brugués, 2019). The two dimensions are not independent – trait anxiety intensifies state anxiety and high trait anxiety predicts high state anxiety (Hamann, 1985; Kenny, 2011; Rumsey, 2015). Numerous studies report significant associations between trait anxiety and MPA (Hamann, 1982; Craske and Craig, 1984; Steptoe and Fidler, 1987; Lehrer et al., 1990; Cox and Kenardy, 1993; Kenny, 2004; Langendörfer et al., 2006; Burin et al., 2019) with some studies concluding trait anxiety is one of the strongest predictors of MPA (Smith and Rickard, 2004; Osborne and Kenny, 2008; Thomas and Nettelbeck, 2014). In their study of MPA and its anxiety correlates, Wiedemann et al. (2021) found Generalized Anxiety Disorder (GAD) to be the strongest predictor of MPA. One study seeking to explain the relationship between trait anxiety and MPA found that lower trait anxiety was positively correlated with participants perceiving their anxiety symptoms as non-detrimental to performance quality (Cox and Kenardy, 1993). Ruggiero (2012) found A-trait to be the only significant predictor of performance difficulties (self-reported), once gender had been controlled for. As well as predicting MPA, A-trait has been found to moderate the interaction between A-state and performance-related behavioral outcomes. Reducing trait anxiety could have real implications for managing MPA.
Cognitive style	Negative cognitions/cognitive style	Cognition arguably forms MPA’s primary component, with negative cognitions predicting MPA more than physiological, emotional or behavioral components (Miller and Chesky, 2004; Kenny, 2006). Studies show significant differences in the cognitions and cognitive styles of highly anxious, compared to low anxious, musicians (Burin and Osório, 2017), with maladaptive cognitive styles contributing to the development and sustenance of MPA (Osborne and Kenny, 2008). The cognitions of anxious musicians can be characterized by recurrent thoughts about negative past performances, imagined avoidance behavior, excessive focus on physiological cues, worrying about not being able to manage physiological arousal or manage negative thoughts, strongly negative self-evaluative focus, irrational beliefs (such as *“I must be perfect”*), expectations of negative evaluation from others, preoccupation with the consequences of suboptimal performances, catastrophizing, inability to manage negative cognitions (self-talk), perceptions of performances as threatening, and general worries about performing (Fehm and Schmidt, 2006; Clark et al., 2014; Kenny et al., 2014; Burin et al., 2019). According to one study (Lehrer et al., 1990), worry is the cognitive factor most significantly correlated with debilitating MPA. The diversion of mental energy to worrying is closely linked to disruption of attention from performance-related tasks (Osborne and Kenny, 2008); distraction and inability to focus are frequently cited as major sources of MPA (Steptoe, 1982; Grindea, 1984; Reubart, 1985; Talbot-Honeck, 1994). Conversely, positive (or realistic) thinking and MPA are negatively correlated (Steptoe and Fidler, 1987; Clark, 1989). Multiple regression analyses of MPA’s correlates showed cognitive factors to be the key predictor of MPA as they underpin all the other factors correlated with MPA in bivariate statistical analysis (catastrophizing, low self-esteem, low self-efficacy, trait anxiety, and maladaptive perfectionism) (Liston et al., 2003).
	Rumination	Negative post-event rumination (PER) is associated with increased MPA levels and decreased enjoyment of performance, with PER decreasing less quickly post-performance in high anxious compared to low anxious participants (Nielsen et al., 2018). Little research has been conducted investigating the relationship between PER and MPA, but these findings are consistent with research on general anxiety and indicate the association between PER and general anxiety.
	Judgmental attitude	Excessive self-criticism in practice and on stage, as well as absolutist judgments regarding one’s behavior, can predict and exacerbate MPA (Lehrer et al., 1990; Sternbach, 2008).
	Catastrophizing	Catastrophizing is a key predictor of MPA (Steptoe and Fidler, 1987; Zinn et al., 2000), with one study finding it to be the single most powerful predicting variable (Liston et al., 2003). While catastrophizing self-statements correlate with high MPA, realistic cognitive self-statements and appraisal of performance correlate with medium levels of MPA (Steptoe and Fidler, 1987), indicating the importance of self-talk.
	Fear of negative evaluation	Fear of negative evaluation (the disapproval of peers, teachers, colleagues, audiences and critics) is a core component of, and significantly predicts, MPA (Papageorgi et al., 2010; Nicholson et al., 2015; Kantor-Martynuska et al., 2018; Burin et al., 2019; Zhukov, 2019). Studies show increased MPA in evaluative versus non-evaluative performing situations (Kobori et al., 2011; Mitchell, 2011), which is problematic given the evaluative nature of professional music-making.
Comorbidities	Psychological issues	Research indicates that musicians’ mental health is an issue of serious concern – studies show that musicians report significantly higher levels of anxiety and depression than the general population (Barbar et al., 2014; Kenny et al., 2014). According to their study of over 2000 UK-based musicians, 71.1% of respondents had experienced panic attacks and/or serious anxiety and 68.5% reported depression (Gross and Musgrave, 2016). The majority of survey respondents ranged between 18–35 years, indicating the scale of the problem among the next generation of musicians. Studies consistently show significant correlations between MPA and a range of psychological challenges including depression, generalized anxiety, stress and substance abuse (alcohol and non-prescription drugs) (Vervainioti and Alexopoulos, 2015; Gross and Musgrave, 2016; Wiedemann et al., 2021). Musicians experiencing high levels of MPA are statistically more likely to experience these comorbid challenges (Burin et al., 2019).
	Physical issues	Research indicates that 55–86% of musicians experience playing-related physical problems severe enough to affect their performance (Kenny et al., 2016; Burin and Osório, 2017; Gembris et al., 2018). These challenges include headaches and stomachaches (Kivimäki and Jokinen, 1994), fatigue and sleep disturbances (Halleland et al., 2009; Dobson, 2011; Voltmer et al., 2012), chronic back pain (Brandfonbrener, 1986), peripheral nerve problems (e.g., focal dystonia) (Schuele and Lederman, 2004), hearing impairment (tinnitus/hearing loss) (Brandfonbrener, 1986; Harper, 2002; Gembris et al., 2018; Topoğlu et al., 2018), and Performance-Related Musculoskeletal Disorders (PMRDs), arising from excessive training, repetition and fatigue and manifesting in pain, numbness, tingling, weakness and other symptoms which disturb high-level performance (Schuele and Lederman, 2004; Voltmer et al., 2012; Vervainioti and Alexopoulos, 2015; Gross and Musgrave, 2016; Topoğlu et al., 2018). The MPA-pain relationship could be explained by performers experiencing psychological distress in response to injuries (Spahn, 2002), that fewer symptoms of pain and fatigue predict higher quality practice and performance (Kreutz et al., 2008; Ginsborg et al., 2009) or that the physiological dimension of MPA causes muscular tension, overstressing the body’s locomotor system causing overuse injuries (Hildebrandt et al., 2012; Rumsey, 2015). Physical challenges are often rated as a cause of MPA by performers (Burin et al., 2019).

Gender (being female) is unequivocally associated with MPA (González et al., 2018; Dobos et al., 2019; Butković et al., 2022), consistent with findings from the general population (de Figueiredo Rocha, 2020). There is also wide agreement that dysfunctional cognitions and cognitive style (rumination, worry, disruptive thoughts, dysfunctional attentional focus, judgmental attitude, catastrophizing and fear of negative evaluation) play a crucial role in MPA’s development and sustenance (Kenny et al., 2014; Lupiáñez et al., 2022). A recent study by Sokoli et al. (2022) investigated the extent to which components of MPA (affective, cognitive and somatic) vary according to factors including practice, musical experience, instrument group, gender and age. Their findings of significant associations between age, gender and instrument group and different MPA components, as well as between specific MPA components (anxious feelings and breathing-related complaints) and decrease in self-reported performance quality have important implications for MPA’s assessment and management (For further investigations of intrapersonal factors associated with MPA’s etiology, see Sadler and Miller, 2010; Burin et al., 2019; Papageorgi, 2020; Wiedemann et al., 2020; Osborne and Kirsner, 2022; Sokoli et al., 2022; Aubry and Küssner, 2023; Kirsner et al., 2023).

As negative personality constructs are associated with MPA, so too are positive ones (Chattin, 2019). Studies have identified several constructs which correlate negatively with MPA including resilience and adaptive coping (Nordin-Bates, 2012; Pecen et al., 2016), openness, conscientiousness and perceived control (Chattin, 2019), positive/realistic cognitive self-statements and performance appraisal (Clark, 1989) and high self-efficacy (González et al., 2018; Sarıkaya and Kurtaslan, 2018; Dempsey and Comeau, 2019). Research is increasingly recognizing the key role of self-compassion in supporting performers across domains – for further discussion, see Baltzell (2016); Kelley and Farley (2019); Lyon and Plisco (2020); Walton et al. (2022). Consistent with the pathological focus of most MPA research, studies largely focus on traits which exacerbate MPA and there is a gap in understanding regarding constructs which protect against MPA. Greater clarity is needed regarding traits and processes which support performers to manage MPA effectively. We turn now to the extrinsic factors associated with MPA’s etiology: Task and Situational factors.

#### 4.2.2. Task

*Task* refers to aspects relating to performance tasks: technical insecurities that cause uncertainty and/or physical tension at the instrument, insufficient preparation, difficulty of repertoire/attempting repertoire beyond one’s ability interpretation of the score, and memorization worries (Kenny et al., 2014; Burin and Osório, 2017; Burin et al., 2019; Ginsborg, 2019). All of these factors are regularly cited by performers as key causes of MPA (Burin et al., 2019). However, they are all highly subjective and thus arguably mediated through *Person.*

#### 4.2.3. Situation

*Situation* can be divided into factors relating to performance mode and to occupational stressors. Within performance mode, solo performance is more associated with MPA than ensemble performance (Brugués, 2019) and public performance and auditions are more associated with MPA than rehearsal or practice (Yoshie et al., 2009; Brugués, 2019). Occupational stressors include the following:

- Social and environmental conditions (Antonini Philippe et al., 2019)
- Financial insecurity (Dobson, 2011; Gross and Musgrave, 2016; Brugués, 2019)
- Job uncertainty (uncertainty about regular employment, highly competitive industry) (Sousa et al., 2016; Brugués, 2019; Burin et al., 2019)
- Demanding work schedule (irregular, unpredictable and anti-social hours, long hours of rehearsal, grueling tour schedules, travel, jetlag) (Burin and Osório, 2017; Brugués, 2019)
- Separation from family (Vervainioti and Alexopoulos, 2015; Sousa et al., 2016; Brugués, 2019)
- Demands of ‘professional sociability’ (especially for freelancers), associated with ‘alcohol as career facilitator’ (Dobson, 2011; Brugués, 2019)
- Environmental challenges (venue): unfamiliar halls, generally unsatisfactory/challenging performance conditions, suboptimal acoustics, temperature, poor seating, lighting and unsafe or cramped backstage, audience characteristics (Parasuraman and Purohit, 2000; Papageorgi et al., 2007)
- Interpersonal challenges: comparison/competition with peers, lack of artistic control/integrity, social tensions (problems with stand partner, back-stabbing between colleagues, lack of confidence in colleagues, issues with management, perceived lack of support from significant others), critical (abusive) verbal style of conductors and frequent changes in leadership (conductor) requiring constant adaptation to different personalities/musical styles (Parasuraman and Purohit, 2000; Burin et al., 2019)
- Performance pressures: scrutiny from audiences, critics & colleagues [managing unrealistic standards of perfection (Kenny, 2004; Dobson, 2011; Burin and Osório, 2017)]

Numerous studies have shown associations between occupational stress and MPA, indicating that they are not independent domains (Voltmer et al., 2012; van Fenema et al., 2013). Based on their qualitative systematic review, Vervainioti and Alexopoulos (2015) concluded that the occupational stressors facing musicians are highly interconnected, forming a complex web between MPA and factors intrinsic and extrinsic to the performer. Across the literature, almost all professional musicians report occupational stress, and many cite it as a key contributor to MPA (Kenny et al., 2014).

While the nature of the association between MPA and situational factors is not entirely clear, there are several plausible links. Given the significance of fear of negative evaluation in predicting MPA, it is unsurprising that performance modes with greater exposure (solo performance and public performance/auditions) are more associated with MPA than those with lower exposure (ensemble performance and rehearsal/practice). Regarding occupational stressors, these are largely beyond performers’ control (Clark et al., 2014), which may contribute to a sense of anxiety. As discussed, all stimuli are filtered through the individual, which could explain why highly anxious musicians are more likely to experience occupational stressors as stressful (Steptoe, 1989). While occupational stressors are inherent to the profession and therefore cannot be removed, protecting musicians from their negative impact will arguably be easier with greater understanding of the mechanisms underpinning the relationship between them and MPA. Seemingly, coping effectively with MPA could reduce the stress of occupational stressors and vice versa. Please see Gross (2015) for discussion of the interaction between emotion regulation, coping and emotional experience.

In sum, MPA is broadly viewed within a multi-factor model, where the degree of anxiety experienced depends on the interaction between a constellation of factors intrinsic and extrinsic to the performer, including biological and demographic variables, personality constructs, and task-based/situational factors associated with musical performance (Antonini Philippe et al., 2022). We turn now how and why MPA manifests, and how it can affect performance and/or performer.

## 5. MPA manifestation and impact

### 5.1. Why MPA manifests

MPA comprises a constellation of partially independent yet interactive responses to perceived threat (Kenny, 2006). In very basic terms, the amygdala constantly scans our environment for threat; when it interprets sensory information as threatening, it triggers the release of stress hormones adrenaline (from the sympathetic nervous system, SNS) and cortisol (via the hypothalamic–pituitary adrenal (HPA) pathway), activating a range of physiological responses which maximize the body’s capacity to deal with the threat (McCarty, 2016). This phenomenon, known as the fight or flight response (Cannon, 1927) can be triggered consciously or unconsciously (Kenny, 2006). Performers vary in their response to anxiety-provoking stimuli for a wide range of reasons including aversive performance experiences (particularly during formative years) (Osborne and Kirsner, 2022) and the intrapersonal factors discussed in Section 4.2.1 which may exacerbate individuals’ perception of a performance as threatening, thus triggering, as well as sustaining, MPA.

The fight or flight response seemingly evolved to protect humans from physical threat endangering their survival. However, the amygdala has not yet evolved to distinguish between physical and psychological threats and triggers the same alarm system in response to both (Kenny, 2011), which is why performing a Mozart concerto can elicit the same physiological response as encountering a tiger. Barlow (2002) differentiates between ‘true alarms’ (automatic physiological activation in response to real danger) and ‘false alarms’ (learned fear responses triggered in the absence of real danger). In the case of true alarms, such as fleeing a burning building, the fight/flight response is vital to survival. However, in the case of false alarms (psychological threats), excessive SNS activity can be problematic, interfering with the fine motor skills necessary for musical performance and potentially creating emotional distress (Kenny, 2009).

Numerous theories have sought to explain what causes so-called ‘false alarms’, including classic operant conditioning (anxiety-conditioning experiences), vicarious (observational) learning, heightened neurobiological hyperreactivity, and psychoanalytic theories, where the audience represents parents or other significant caregivers (Kenny, 2009). While no account is comprehensive, and causes are most likely multi-determined (Kenny, 2009), it seems clear that a prerequisite for MPA to manifest is the performer’s initial perception of a stimulus (performance) as threatening (Osborne and McPherson, 2019).

### 5.2. How MPA manifests

#### 5.2.1. Physiology

Physiological responses to perceived threat include increased cardiovascular activity (tachycardia, palpitations and increased blood pressure), respiratory changes (hyperventilation, difficulty controlling breathing, and shortness of breath), dry mouth, throat constriction (difficulty swallowing), blurred vision, perspiration and clamminess, hot or cold flushes, dizziness, urinary urgency, gastrointestinal activity (butterflies, nausea, vomiting, upset stomach, diarrhea), excessive muscle tension and fatigue, reduced motor control, coordination and agility, muscle spasms/tremor, transient limb paralysis, uncontrollable shaking, numbness or tingling in extremities and hormonal changes (Spahn, 2015; Altenmüller and Ioannou, 2016; Guyon et al., 2020; Turan et al., 2022). Excessive muscular tension is particularly problematic as it can disrupt the fine motor control required for performance and increase risk of PMRDs (Yoshie et al., 2009; Pell, 2020; Vivas et al., 2021). Given the unpleasantness of these experiences, it is unsurprising that controlling them is often the most urgent goal for performers (Zhukov, 2019).

Although physiological data are often used to assess MPA level, evidence to support their use as a proxy for MPA is equivocal (Yoshie et al., 2009). Indeed, research shows that performers with significantly elevated heart rate and blood pressure can report low MPA while performers showing normal physiological parameters can report intense MPA and that physiological arousal often correlates positively with optimal performance (Craske and Craig, 1984; Spahn et al., 2010; Endo et al., 2014; Studer et al., 2014). These findings suggest that physiological activation seemingly becomes problematic only when met with/experienced alongside psychological distress – the importance of negative cognitive appraisal in generating and exacerbating MPA arguably cannot be understated (Steptoe, 2001; Osborne and Kenny, 2008). We therefore turn now to the psychological components of MPA.

#### 5.2.2. Cognitions

Research consistently indicates the prominence of dysfunctional cognitions in highly anxious performers (Kenny and Osborne, 2006; Sokoli et al., 2022). While cognitions are generally discussed as one dimension, it may be helpful to divide them into two discrete (albeit somewhat overlapping) categories: cognitive content (specific worries) and cognitive style (type of thinking). *Cognitive content* (worries), centered on the perceived threat of risk or danger (Salmon, 1990) include worrying about memory lapses, errors, technical or musical incompetence, negative evaluation, failure, embarrassment or humiliation, and inability to control the effects of physiological arousal (McGrath, 2012; Fernholz et al., 2019). Although cognitions are often discussed as a response to physiological activation, they can also be part of the initial anxiety response – anxiety distorts cognitive style and constricts thinking, manifesting in a maladaptive *cognitive style* characterized by irrational and self-defeating failure-focused cognitive patterns (Osborne and Franklin, 2002; Altenmüller and Ioannou, 2016).

A fundamental aspect of MPA seems to be the disruption of task-oriented cognitions – Steptoe (2001) identifies three key types of disruption: (1) catastrophizing (exaggerating the probability and impact of negative/disastrous events in performance) which is strongly positively correlated with MPA (Valentine, 2002), (2) preoccupation with possible negative evaluation, and (3) heightened perception of physiological changes and interpreting them as indicating loss of control and inevitable collapse. Other maladaptive cognitive styles include negative self-talk (excessive focus on perceived inadequacies), irrational beliefs (such as conflating one’s performance with one’s self-worth), excessive focus on task-irrelevant thoughts provoked by internal or external distractions and rumination (self-doubts, negative and judgmental thoughts about oneself and one’s performance, projected criticism from the audience and excessive focus on technical inadequacies) (Perdomo-Guevara, 2014; Spahn, 2015; Zhukov, 2019). Anxious cognitions can impair concentration and focus, drain self-confidence and interfere in the creative process (McGrath, 2012; Patson and Loughlan, 2014). *In extremis*, negative post-performance rumination can resemble post-traumatic stress disorder (Sternbach, 1993).

#### 5.2.3. Emotions

The fundamental experience of anxiety is arguably anxious apprehension– a future-based state characterized by feelings of helplessness based on the sense that one can neither predict nor control possible threats (Kenny, 2009). Other commonly reported emotions associated with MPA include dread, fear, panic, terror, stress, insecurity, irritability, anger, moodiness, embarrassment, denial, depression, distress, shame, frustration and guilt (McGrath, 2012; Spahn, 2015; Kantor-Martynuska et al., 2018; Fernholz et al., 2019).

#### 5.2.4. Behaviors

Behavioral responses encompass a range of conscious and unconscious behaviors such as overt physical expressions of anxiety (alterations in body language such as postural distortions, tensed or hunched shoulders and distressed or deadpan facial expressions) (Patson and Loughlan, 2014), isolation from others (Roland, 1994), avoidance behaviors (Lederman, 1999; Fernholz et al., 2019) such as avoiding practicing difficult technical passages (Lehrer and Woolfolk, 1982), or performance avoidance (Salmon, 1990), and general unrest (agitation, fidgetiness, escapist tendencies) (Steptoe, 2001; Spahn, 2015). Performers may also engage in ‘safety behaviours’ such as alcohol or drug consumption, distraction techniques (Fernholz et al., 2019) and compulsive, ritualized, behaviors such as repetitive practice, repeatedly checking instrument or moistening lips (Patson and Loughlan, 2014; Spahn, 2015; Altenmüller and Ioannou, 2016). It is unclear whether these behaviors are manifestations of MPA, coping strategies to manage MPA, or both. We turn now to MPA’s impact on performance quality, an issue of paramount importance to musicians (Kenny, 2011).

### 5.3. How does MPA impact performance quality?

Numerous theories have sought to explain the complex relationship between anxiety (or arousal) and performance quality across a range of domains. The first, and most widely used, is the Yerkes and Dodson (1908) ‘Inverted U’, which posits that performance quality is highest when arousal is moderate; below or above this optimal functioning zone, performance quality decreases (Williamon, 2004). Although this relationship seems well-established, it only considers physiological arousal, rendering it overly simplistic. Fazey and Hardy’s (1988) Catastrophe Theory adds a third dimension – cognition – arguing that the Yerkes-Dodson Law holds when cognitive anxiety is low, but when cognitive anxiety is high, the quality of performance can deteriorate catastrophically (Hardy and Parfitt, 1991). For detailed discussion of the Yerkes-Dodson and Catastrophe Models across performance domains, see Ruggiero (2012).

The last two decades have seen the development of models which focus specifically on the relationship between MPA (as opposed to arousal/performance anxiety in other domains) and performance quality (Zinn et al., 2000; Kirchner, 2003; Papageorgi et al., 2007; Chow and Mercado, 2020; Osborne and Kirsner, 2022). Collectively, these five models, summarized in Table 4, illuminate different aspects of the complex and multidimensional relationship between MPA and performance quality and contribute significantly to current understanding. We will now suggest four areas for possible further exploration.

**Table 4:** Models exploring how MPA disrupts performance.

Zinn et al. (2000)	Zinn et al.’s (2000) Psychophysiological MPA model attributes MPA to physiological manifestation of repressed anxiety; performance quality is impaired through errors caused by SNS overactivation.
Kirchner (2003)	Based on her qualitative study investigating MPA in solo pianists, Kirchner (2003) proposes a model wherein MPA is initially triggered by threat, and then manifests in cognitive, emotional and physiological responses which interact, both with each other, and with the performer’s identity. This model clearly shows the circularity of MPA – each domain interacts with the others, rendering the cycle difficult to stop once in motion.
Papageorgi et al. (2007)	Papageorgi et al. (2007) offer a conceptual framework of MPA, which maps out comprehensively out the different temporal stages of MPA’s trajectory, from intrapersonal, task and environmental factors, to the performer’s evaluation of the performance context, through the performance to post-performance conditions.
Chow and Mercado (2020)	Chow and Mercado (2020) critique existing psychological MPA theories based on an overreliance on relating a performer’s physiological and psychological state to their capacity to maintain focus and execute learned skills, without accounting for how past experience and task-specific expertise moderate the intensity of anxiety experienced in performance. The authors propose a Connectionist Model in which MPA is co-determined by experience-dependent plasticity, cognitive and physiological states and competition between motivational systems. The model offers valuable insight into how experience-dependent plasticity may contribute to the development of socio-evaluative anxiety in pressurized situations.
Osborne and Kirsner (2022)	The most recent MPA model (Osborne and Kirsner, 2022) illustrates how relevant past experiences interacting with maladaptive schemas and low self-efficacy to distort a musician’s perception of the likelihood and consequences of negative evaluation in highly evaluative performance situations. In performance, physiological and attentional changes interact to produce performance problems, which can either lead to coping strategies enabling the performer to re-engage in performance, or to disengagement or avoidance.

#### 5.3.1. Performers who experience MPA yet do not demonstrate performance problems

The Yerkes-Dodson ‘inverted U’ has underpinned academic discussion of anxiety’s impact on performance quality across domains for over a century, leading to the widely held perception that performance quality deteriorates beyond a certain level of anxiety. However, evidence to support this is not clear-cut (Osborne et al., 2020). Despite evident cognitive differences between low and high anxious performers, highly anxious performers do not necessarily display impaired performance (Kenny, 2004; Kantor-Martynuska et al., 2018). In their study of professional orchestral musicians, Kenny et al. (2014) found that even the most highly anxious performers rarely experienced performance catastrophes, or indeed any significant impairment of performance quality. Indeed, numerous renowned artists (including Pablo Casals, Steven Osborne, Vladimir Horowitz, Maria Callas and Andrea Bocelli) have publicly disclosed their personal struggles with MPA, while maintaining highly successful musical careers. From their continued critical acclaim, one can reasonably infer that they managed to perform to a consistently high level, despite self-reported debilitating anxiety, seemingly refuting the premise that excessive arousal causes performance deterioration.

It seems that musicians can perform to high levels of excellence while experiencing significant levels of MPA, but that their wellbeing and enjoyment of performance can still suffer (Kenny, 2011). This suggests that (1) existing models account for MPA’s impact on performance, but not performer, and (2) that further research is needed to understand which variables or processes moderate the relationship between MPA and performance quality.

#### 5.3.2. The interaction between MPA responses

Only one of the models (Kirchner, 2003) unpacks the interaction of MPA responses. As cognitions underlie behaviors and interact reciprocally with emotions and physiology (Valentine, 2002; Perdomo-Guevara, 2014), negative cognitions can trigger interaction between all four domains, creating a vicious circle whereby responses ‘feed off’ each other and can become overwhelming and difficult to manage (De Felice, 2004; Oyan, 2006; Kenny, 2011). Wolfe (1989) offers the example of a flutist experiencing dry mouth, whose worries about dry mouth exacerbate dryness and increase anxiety, in turn exacerbating dry mouth and so on. Seemingly, the interaction *between* responses may offer a more useful way to understand their impact than their *presence.* While interaction between MPA responses seems key to understanding its impact, the relational aspect of responses is under researched (Stanson, 2019) and prevailing theories are not addressing adequately how interaction works, or how it can be effectively managed to mitigate its impact on performance.

#### 5.3.3. Performers’ interpretations of MPA

Research in both music and sport indicates that anxiety is multidimensional, with intensity and direction of symptoms forming distinct constructs (Jones and Swain, 1992; Mor et al., 1995; Miller and Chesky, 2004). Growing evidence suggests that performers’ interpretation (cognitive appraisal) of anxiety impacts performance significantly more than its presence (Osborne and Kenny, 2008; Clark et al., 2014; Osborne and McPherson, 2019; Osborne and Kirsner, 2022), with facilitative interpretations enhancing performance and debilitative interpretations leading to decreased enjoyment of performing and deleterious effects on performance quality (Yoshie et al., 2009). Performers’ interpretation of MPA as positive or negative is seemingly mediated by a range of factors including performing experiences, task mastery, self-efficacy and perceived control over anxiety symptoms (Osborne and Kirsner, 2022).

Research investigating performers’ interpretations of MPA has largely focused on the positive/negative binary. However, acceptance (versus resistance) may offer an alternative framework to understand performers’ interpretations of MPA, and its impact on performance. Anxiety-related psychopathology is characterized by experiential avoidance of fear (Schanche et al., 2020), whereas psychological acceptance correlates positively with improved coping strategies (Baer, 2003) and negatively with emotional dysregulation and other indices of poor mental health (Hitchcock et al., 2016). The consistent findings that performers’ response to MPA has greater impact than its presence has important implications for understanding how MPA can impact performance and the ‘response’ stage should arguably be a critical component in MPA models.

#### 5.3.4. MPA and attentional focus

*“My experience is what I attend to”* [William James, cited in Tremayne and Morgan (2016), p. 389].

Across domains, optimal performance demands intense concentration and focus (Talbot-Honeck, 1994). Indeed, within musical performance the centrality of attention regulation has long been recognized (Rife et al., 2000; Kenny, 2011). One of MPA’s most common and challenging effects is attentional changes – both the narrowing of focus (Valentine, 2002) and the shift in focus from task-relevant to task-irrelevant, manifesting in negative self-evaluation, worries, rumination and preoccupation with physiological changes (Liston et al., 2003; Kenny, 2011). Essentially, anxiety hijacks attention from the music (Salmon and Meyer, 1992; Oyan, 2006). Humans have finite attentional capacity (Kantor-Martynuska et al., 2018) – when attention is both constricted by anxiety and depleted attentional resources are hijacked by negative cognitions, there is insufficient bandwidth to focus on task-relevant processes, which can impair performance, in turn exacerbating physiological, cognitive and emotional responses (Kenny, 2009).

Attentional Control Theory (ACT, Eysenck et al., 2007) offers a compelling account of the complex relationship between attentional focus and performance quality. According to this extramusical theory, anxiety decreases attentional control and processing efficiency by overloading working memory with anxious cognitions and increased focus on threat-based stimuli, thus impairing executive function. Eysenck and Calvo (1992) and Eysenck et al. (2007) differentiate between effectiveness and efficiency in performance: *effectiveness* refers to performance quality, and seems independent of anxiety, while *efficiency* (the interaction between effectiveness and deployment of personal resources) is impaired by anxiety as negative cognitions disturb task-relevant processing. When anxiety induces performers to deploy compensatory strategies/resources, performance effectiveness (quality) overall will not be impaired.

This finding is significant as it arguably explains how experience and expertise can mediate MPA’s impact on performance quality – the elite performers discussed above may have sufficient resources to enhance their efficiency, thus maintaining high levels of performance effectiveness. This may also explain why performers can perform to very high levels, but at a high personal cost in terms of their wellbeing and reduced enjoyment of performance. Studies have been conducted using ACT to predict anxiety-related performance outcomes across domains cognitive performance (Eysenck et al., 2007), athletics (Causer et al., 2011) and music performance (Ruggiero, 2012). A study by Oudejans et al. (2017) found that music students experienced a significant increase in self-reported negative cognitions immediately before choking (crumbling under pressure), suggesting that anxiety-induced attentional changes may cause performance deterioration in less expert/experienced performers. In their study comparing external versus internal focus conditions, Mornell and Wulf (2019) found increased musical expression and technical accuracy in the external focus group. Collectively, these studies suggest that planning and training particular attentional foci could optimize learning and performance outcomes. Further research applying ACT to musical performance could offer valuable insight into the different constructs of effectiveness and efficiency, and help performers navigate the challenges associated with reduced bandwidth under pressure.

In sum, we suggest that developing a comprehensive MPA model requires further insight into (1) understanding performers who experience MPA yet do not demonstrate performance problems, (2) the nature of the interaction between MPA responses, (3) how performers’ interpretation of their experience impacts performance and (4) the relationship between attentional changes and performance quality/experience. We turn now to the ways in which musicians seek to manage MPA.

## 6. Managing MPA

Given the myriad ways MPA can threaten performance, it is unsurprising that musicians utilize a range of strategies to try and mitigate its impact. This section will discuss the two routes musicians take to manage MPA: Coping strategies and interventions. Coping here encompasses the conscious/unconscious application of behaviors/strategies musicians employ to manage MPA; interventions here are more formalized programs based on the explicit training of specific strategies to manage MPA, delivered by a trained practitioner. As these two topics are qualitatively different, they will be presented separately.

### 6.1. Coping with MPA

Coping is defined as the “constantly changing cognitive and behavioral efforts to manage specific external and/or internal demands that are appraised as taxing or exceeding the resources of the person” (Lazarus and Folkman, 1984, p. 141). There is broad consensus that it is not the type or quantity of stress that determines its impact, but how the individual copes with stress (Lazarus, 2006). While there is agreement regarding what coping is, the models used to categorize coping strategies vary across the literature. Distinctions include adaptive versus maladaptive, effective versus ineffective (Wolfe, 1990), problem-orientated versus emotion-orientated (Studer et al., 2011), appraisal versus avoidance (Langendörfer et al., 2006), cognitive versus behavioral (Burin and Osório, 2017), and trait versus state (Langendörfer et al., 2006). The aim of this discussion is not to classify musicians’ coping strategies using theoretical models, but rather to offer an overview of the coping strategies musicians use, and their relationship with MPA.

Over the past 35 years, numerous researchers have investigated the coping strategies musicians use to manage MPA. Studies range in methodology (both qualitative and quantitative), population (conservatoire and university students, amateur and professional, classical and jazz) and location, including: the UK (Steptoe and Fidler, 1987; Steptoe, 1989), North America (Wolfe, 1990; MacAfee and Comeau, 2022), Australia (Roland, 1994; Kenny et al., 2014), Italy (Biasutti and Concina, 2014), Spain (Hernández et al., 2018; Lupiáñez et al., 2022), Switzerland (Studer et al., 2011), Estonia (Kiik-Salupere and Ross, 2020), Turkey (Cornett and Urhan, 2021; Yadigaroğlu, 2021), Malaysia (Zakaria et al., 2013), Taiwan (Huang and Song, 2021) and Brazil (Burin et al., 2019). Across these studies, the strategies most commonly used to cope with MPA include positive self-talk, meditation, distraction and social support, relaxation exercises (muscle relaxation and deep breathing), increased practice, mock performance practice, ritualized behaviors and substances including beta blockers, alcohol and illicit drugs. Use of coping strategies varied between students and professionals, by gender, and by nationality (prayer was included in the Malaysian and Turkish studies, indicating the importance of cultural context).

There is a complex and seemingly paradoxical relationship between coping and MPA in that the two constructs are positively correlated – greater use of coping is associated with increased MPA (Steptoe, 2001). There are a number of possible explanations for this. Firstly, more anxious musicians are more likely to engage in deliberate coping behaviors which may prove effective, but MPA levels remain higher than their nonanxious colleagues (Steptoe and Fidler, 1987; Lehrer et al., 1990). Secondly, strategies deployed to alleviate MPA may be counterproductive (Langendörfer et al., 2006): Studies investigating specific coping strategies show positive correlations between substance use and MPA (Park, 2010) and dysfunctional coping (social support and avoidance strategies) and MPA (Biasutti and Concina, 2014). Thirdly, coping is a multidimensional construct with both adaptive and maladaptive components. Effective (adaptive) coping is associated with beneficial physiological states, reduced negative affect, perceiving anxiety as facilitative and adaptive MPA (Wolfe, 1990; Langendörfer et al., 2006; Papageorgi et al., 2007; Park, 2010). Conversely, inadequate (maladaptive) coping is significantly positively correlated with, and significantly predicts, MPA (Sinden, 1999; Langendörfer et al., 2006; van Fenema et al., 2013; Thomas and Nettelbeck, 2014). Lastly, coping is highly individual – the same strategies can be effective or ineffective depending on the individual, and indeed, strategies used by an individual may vary in efficacy across different performance contexts (Burin and Osório, 2017). For further discussion on the relationship between coping and MPA, see McNeil et al. (2022).

Researchers agree that effective coping is vital to managing MPA (Spahn et al., 2016; Osborne and McPherson, 2019). However, there remain gaps in current understanding of the relationship between coping and MPA: what mediates the efficacy of different coping strategies? Are some ‘better’ than others, or does their efficacy depend wholly on the individual? Are strategies more effective when combined? One interesting omission across these studies concerns what, exactly, musicians are trying to achieve through their coping efforts. If their aim is to eliminate MPA, this may be unrealistic, even undesirable, given the necessity of arousal for optimal performance, thus rendering the coping strategies ineffective. Arguably, being explicit about, and possibly reframing, what exactly the coping strategies are being used for may offer a different perspective on their efficacy.

Problematically, there remains little consensus regarding what actually constitutes effective coping. There are two main possible reasons for this ambiguity. Firstly, as discussed, coping is highly individual. Secondly, musical performance is largely researched through a pathological lens, focusing on musicians who experience debilitating MPA and therefore arguably cope inadequately. The few studies which have investigated the psychological characteristics of performers through a non-pathological lens found flexibility of coping and the deployment of a range of effective coping strategies (including positive anxiety appraisal and adaptive lifestyle habits) to be key (MacNamara et al., 2008; Pecen et al., 2018). Despite the significance of coping in predicting and sustaining, or protecting against, MPA, the complex interaction between the two constructs has not yet been studied sufficiently (Hernández et al., 2018).

Of particular concern is the prevalent use of substances to manage MPA (Kenny et al., 2014; Hernández et al., 2018; Burin et al., 2019). Alcohol impairs fine-motor skills, reaction time, memory and coordination, all vital for performance, and can lead to addiction and serious health issues (Zhukov, 2019). In Burin et al.’s (2019) study of 240 Brazilian musicians, 25% of participants who used alcohol to manage MPA showed signs of alcohol abuse. Beta-blockers can also have problematic side-effects and become psychologically addictive (Studer et al., 2011). Based on their study of Australian orchestral musicians, Kenny et al. (2014) reported that 30% of participants rely on beta-blockers to manage their anxiety. Indeed, the British Association of Performing Arts Medicine suggest beta blockers and tranquilizers as MPA management options on their website [British Association for Performing Arts Medicnie (BAPAM), n.d.], which suggests (1) that we are urgently in need of healthy and effective solutions to manage MPA and (2) the extent to which the music profession has normalized the use of medical substances to manage anxiety. To be clear, this is *not in any way* a moral crusade, but with so many musicians at both student and professional level, relying on substances to perform, it seems clear that musicians are experiencing real stress in large numbers and lack adequate coping strategies to manage it.

In sum, while musicians clearly utilize a range of coping strategies, these vary significantly in terms of healthfulness – ranging from healthy to destructive (Helding, 2016) – and efficacy, with significant numbers of musicians employing largely ineffective strategies (Kenny, 2005; Burin et al., 2019). Studies frequently report a disparity between the percentage of musicians using a particular strategy, and the percentage rating that strategy as being effective, indicating a lack of knowledge regarding which strategies actually help. Research suggests that in the absence of guidance regarding effective coping, musicians are likely to engage in coping mechanisms which may inadvertently exacerbate MPA (including seeking social support and avoidance) (Biasutti and Concina, 2014), whereas those who are taught effective coping strategies learn to manage the effects of MPA on performance (Juncos et al., 2017; Osborne and McPherson, 2019). Given its crucial role, it is concerning that so many musicians report limited coping skills. We turn now to available MPA interventions.

### 6.2. MPA interventions

In the 50 years since the first documented MPA intervention (Wardle, 1969), a plethora of interventions have emerged from a diverse range of theoretical perspectives. These include pharmacotherapy, psychodynamic and psychoanalytic therapies, cognitive, behavioral and cognitive-behavioral therapies, mindfulness-and-acceptance-based approaches, performance coaching, multimodal interventions, virtual reality and exposure therapies, among others. As a comprehensive review of available MPA interventions is beyond the scope of this review, Table 5 defines the major types of intervention, discusses their theoretical underpinnings and signposts the reader to empirical studies investigating their efficacy. There is a curious tension between individual intervention studies and prevalence rates: Studies broadly report statistically significant change at the *granular* level, yet this change is not seen at the *population* level. MPA’s unchanged prevalence indicates that current approaches are not having a meaningful impact overall. This section will review the current state of play of intervention research, from methodological and conceptual perspectives.

**Table 5:** Definitions and theoretical underpinnings of MPA interventions with signposting to relevant empirical studies.

Intervention	Overview/Definition/Theoretical underpinnings
Pharmacotherapy	Physiological psychologists conceptualize MPA as a predominantly physiological phenomenon: the perception of threat activates the fight/flight response and the excessive adrenalin released causes physiological changes which can impair performance (Spahn, 2015). Based on this perspective, pharmacotherapy has been proposed as an effective solution to managing MPA (Nagel, 1990). Of the pharmacological treatments available, beta blockers are the most widely used, and widely investigated (Spahn, 2015). Beta blockers (beta-adrenergic blocking agents) are a group of medications designed to treat heart conditions by lowering blood pressure through blocking the impact of adrenalin. They are commonly used as a pharmacological treatment of MPA as they ameliorate some physiological symptoms, including tremor, reduced motor coordination, increased heart rate and palpitations (McGrath, 2012). Although in the early 1980s, several clinicians proposed beta-blockers as an effective solution to MPA (Neftel et al., 1982; James and Savage, 1983; Brandfonbrener, 1990), their use today is controversial, with ongoing debates regarding their efficacy and safety. Large-scale studies investigating the prevalence of beta-blocker use (Fishbein et al., 1988; Lockwood, 1989; Kenny et al., 2014) report 20–30% of professional orchestral musicians take beta-blockers to manage MPA. Anecdotal evidence suggests these figures may actually be significantly higher (Kenny, 2011; Patson and Loughlan, 2014). Concerningly, many of these users are unprescribed, and therefore without medical supervision. The use of beta-blockers remains controversial, with a lack of clarity regarding their efficacy, and more concerningly, their safety. Although they may be effective in reducing the physiological symptoms of MPA, they do not address the psychological components of MPA (Lehrer, 1987) and there are serious concerns associated with their use, especially without medical supervision (Kenny et al., 2014). Given the high levels of usage among professional musicians, and the lack of unanimous opinion regarding their safety (Lederman, 1999), there is an urgent need for approaches which tackle MPA effectively, thus reducing the risk to the health and wellbeing of musicians for whom unprescribed medication seems the only option. Current, methodologically rigorous, studies investigating beta-blockers are not possible due to ethical reasons. Furthermore, their use is associated with risks including psychological dependence, clinical side effects, compromised performance quality and increased anxiety (Brantigan et al., 1979; James and Savage, 1983; Lederman, 1999; McGinnis and Milling, 2005; Burin and Osório, 2017). The continued prevalence of beta blockers (based on both empirical and anecdotal evidence) indicates that there is no viable alternative.
Psychodynamic/psychoanalytic therapies	According to psychoanalytic theory, MPA stems from early life experiences (such as lack of secure attachment with significant caregivers) and expresses unconscious conflicts and defence mechanisms (Spahn, 2015). Based on this theoretical basis, psychoanalytic and psychodynamic therapists seek to enable performers to understand, and thus resolve, the conscious and unconscious conflicts associated with performing (Kenny, 2011). Proponents of this approach further argue that debilitating MPA may offer secondary gains, such as increased attention and care from significant others, or the avoidance of success which may pose greater psychological threat than failure (Sataloff et al., 1999). Only two case reports have investigated the use of psychoanalytic/psychodynamic therapy for MPA (Safirstein, 1962; Kenny et al., 2016). Although both studies reported significantly reduced MPA, they both employed a single-participant design, precluding generalizability. There is currently minimal empirical support for a psychodynamic approach (only two participants, five decades apart). Further research is needed to support the efficacy of this approach.
Cognitive therapies	Cognitive theories emphasize the impact of cognitions on physiological and behavioral symptoms, and argue that as humans have limited attentional resources, how attention is directed will impact task performance (Kenny, 2011; Spahn, 2015). Based on this theoretical premise, cognitive therapies seek to modify irrational or dysfunctional thought patterns through cognitive restructuring, a process of replacing negative, unhelpful, or catastrophic thinking with rational and constructive ways of thinking (Kenny, 2011). Several studies have linked MPA with dysfunctional cognitive processes, including catastrophizing and maladaptive perfectionism (Mor et al., 1995; McGinnis and Milling, 2005; Spahn, 2015), justifying the use of cognitive therapy to manage MPA. Although a number of studies have emphasized the role of negative cognitions in maintaining MPA (Steptoe and Fidler, 1987; Osborne and Franklin, 2002), very few studies have investigated the effects of cognitive therapies alone on MPA. (See Kendrick et al., 1982; Sweeney and Horan, 1982). Evidence to support cognitive therapies is equivocal – there is a lack of consistent evidence to support cognitive therapy as an independent modality (McGrath, 2012). Brugués (2019) posits that no conclusions can currently be drawn regarding the efficacy of cognitive interventions due to insufficient evidence resulting from a dearth of studies. A greater number of studies is needed to build a robust evidence base.
Behavioral therapies	Behavioral interventions encompass those which seek to modify behaviors in order to modify one’s state, including relaxation training and exposure therapies, and to alter dysfunctional behaviors which occur in response to anxiety (Brugués, 2019). Relaxation techniques focus on reducing the physiological symptoms of anxiety from one of two angles: ‘mind to muscle’ or ‘muscle to mind’ (Harris, 1986). ‘Mind’ strategies include visualization, while ‘muscle’ strategies include physical relaxation techniques such as stretching, or Progressive Muscular Relaxation (PMR) (McGrath, 2012). Exposure therapy, whether real or virtual, involves repeated, or graded, exposure to the feared stimulus in the absence of danger, so as to overcome anxiety (Wardle, 1969; Appel, 1976; Kendrick et al., 1982; Sweeney and Horan, 1982; Kim, 2008; McGrath, 2012; Bissonnette et al., 2015; Zyl, 2021; Osborne et al., 2022; Bellinger et al., 2023; Candia et al., 2023) While individually effective, the studies cannot be used in totality to form an evidence base as each study used entirely different treatment approaches: insight-relaxation, systematic desensitization, behavioral rehearsal, cue-controlled relaxation, breathing exercises, free improvisation, and virtual exposure therapy. Although individual studies report positive results, the heterogeneity of intervention modalities, assessment and outcome measures, and sampling strategies (too small/diverse) preclude firm conclusions regarding the efficacy of any one approach within behavioral interventions (Kenny, 2011; McGrath, 2012; Brugués, 2019). Behavioral interventions appear effective, but the heterogeneity of intervention modalities preclude firm conclusions regarding the efficacy of any one approach (Brugués, 2019).
Cognitive-behavioral therapy (CBT)	Emerging from the separate domains of cognitive therapy and behavioral therapy, CBT is based on the premise that emotions and behaviors are largely informed by cognitions – thoughts, ideas or beliefs about oneself and others; as well as impacting emotions and behaviors, cognitive processes which are irrational and self-defeating may cause, or intensify, physiological stress reactions (Kenny, 2011). CBT psychology largely attributes the sources of MPA to performers’ recurrent cognitive patterns and attitudes (McGrath, 2012). CBT interventions are systematic, goal-oriented processes which teach participants to identify, evaluate and challenge (through modifying or replacing) maladaptive cognitive and behavioral patterns (Brugués, 2011a; McGrath, 2012; Spahn, 2015). At first glance, the evidence base for CBT as an effective intervention for MPA appears broad and compelling. Indeed, several studies report significant reductions in MPA following a CBT intervention (Clark and Agras, 1991; Osborne et al., 2007; Braden et al., 2015). However, the overall picture is more complex than it initially seems. In their systematic review of MPA interventions, Burin and de Lima Osório (2016) included six CBT studies. While Osborne et al. (2007) and Braden et al. (2015) both report significantly decreased MPA in adolescent music students, the other four studies are not CBT interventions. Bien Aime (2011) explicitly criticizes CBT and uses an approach called Solution-Focused Brief Therapy (SFBT), Errico (2012) uses a ‘researcher-designed intervention’ with no mention of CBT and both Clark and Williamon (2011) and Hoffman and Hanrahan (2012) investigate PST interventions, not CBT. Fernholz et al.’s (2019) systematic review tells a similar story. Of the 10 CBT studies reviewed, all indicated positive impact on MPA. However, six are not CBT interventions. Juncos and Markman (2016), Juncos et al. (2017) investigate Acceptance and Commitment Therapy (ACT), a method which they explicitly differentiate from CBT in terms of its theoretical underpinnings and treatment approach. Similarly, Lazarus and Abramovitz (2004) explicitly differentiate multimodal training (which they investigate in this study) from CBT. Bissonnette et al. (2015) investigate virtual reality exposure training, with no cognitive restructuring component. Nagel et al. (1989) combine cognitive therapy with relaxation and biofeedback training, with no attempt to isolate the specific impact of the CBT components. Rider (1987) describes his intervention as primarily music therapy, incorporating aspects of CBT alongside other treatment modalities. Of the remaining four studies, two use a single subject design (Norton et al., 1978; Salmon, 1992), precluding any generalizability. Sweeney and Horan (1982) (*n* = 49 undergraduate music students) compared CBT with cognitive and behavioral interventions, with only a fifth of participants in the CBT group, and concluded that it was no more effective than the single modalities. Brodsky and Sloboda (1997) (*n* = 54 orchestral musicians) assessed three treatment conditions: traditional psychotherapeutic counseling, counseling plus music, or counseling plus music and vibrotactile sensations. While treatment conditions were loosely based on a CBT approach, this study does not strictly adhere to a CBT treatment model. Cognitive-behavioral therapies are surprisingly difficult to evaluate as most of the CBT interventions included in reviews are in fact not CBT. Due to the limited number of studies on CBT as a single intervention and methodological weaknesses across studies, positive results require cautious interpretation, and conclusions cannot be drawn regarding its efficacy (Burin, and de lim A Osóri O, F., 2016). One major obstacle to ascertaining the efficacy of CBT is the lack of clarity between cognitive therapies, behavioral therapies and cognitive behavioral therapies. For example, Kendrick et al. (1982) is regularly cited as a key CBT study, yet the three treatment conditions are cognitive OR behavioral, or control group. Kenny (2011) argues that these distinctions are somewhat arbitrary because in humans, as sentient beings, behavior cannot occur without a cognitive component. Much greater conceptual clarity is needed to delineate clearly between these modalities. While CBT is regularly hailed as the gold standard in anxiety treatment, little evidence suggests it is superior to either cognitive or behavioral techniques in isolation (McGinnis and Milling, 2005). Minimal engagement with the mediators and moderators results in a lack of clarity on how the components of CBT effect change (McGinnis and Milling, 2005). A lack of longitudinal studies preclude conclusion regarding CBT’s long-term benefits (Kenny, 2011) and further investigation is needed, with larger samples and adequate control groups (Nagel, 1990). Due to the limited number of studies on CBT as a single intervention and methodological weaknesses across studies, positive results require cautious interpretation, and conclusions cannot be drawn regarding its efficacy (Burin, and de lim A Osóri O, F., 2016). Conceptually, CBT may be problematic as it may increase performers’ explicit-monitoring of cognitions which could interfere with the automatic processes and motor skills required for performance (Farnsworth-Grodd, 2012). See Faur et al. (2022) for a meta-analysis of CBT interventions for MPA.
Performance coaching, psychological skills training and multimodal interventions	PST encompasses systematic practice of the psychological skills required for optimal performance, including self-regulation, motivation, imagery, goal setting, confidence, concentration and arousal management (Ford and Arvinen-Barrow, 2019). Based on the conceptualization of MPA as a complex, multi-multidimensional phenomenon, multimodal therapies combine various treatment modalities to target all aspects of MPA from an individualized person- and problem-oriented approach (Spahn, 2015). Modalities include psychodynamic therapy, CBT strategies, autogenic training, body awareness, mental techniques, imaginative techniques, breathing exercises, concentrative exercises, preparation techniques, performance training, cognitive strategies, video-feedback. Psychological/Mental Skills Training (PST/MST) has been used to enhance performance in athletes for over 50 years and has recently been investigated with musicians (see Lazarus and Abramovitz, 2004; Clark and Williamon, 2011; Osborne et al., 2014; Finch and Moscovitch, 2016; Hatfield, 2016; Spahn et al., 2016; Cohen and Bodner, 2019; Pecen, 2019; Finch and Oakman, 2022; Logan, 2022) Interestingly, in their systematic review of PST studies for musicians, Ford and Arvinen-Barrow (2019) include several interventions which are not labeled as PST anywhere else in relevant literature, including meditation, yoga and Alexander Technique. While several studies report a range of benefits, the authors criticized the disparate theoretical and empirical frameworks employed to underpin the interventions, as well as interventions’ inconsistencies across length, frequency and dosage. Aside from the very small sample sizes which preclude generalizability, multimodal interventions are, by definition, highly complex, requiring interrogation into the impact of individual modalities (not included in any cited study). Furthermore, the complexity of a highly individualized (custom-made) approach precludes accessibility. Multimodal interventions and Psychological Skills Training appear highly effective, but their heterogeneity in terms of intervention design (components) preclude firm conclusions being drawn. Overall, PST interventions for musicians appear highly promising, but research is still in the early stages (Matei and Ginsborg, 2017) and there is a need for methodological homogeneity across studies to build a robust evidence base.
Meditation, mindfulness-and-acceptance-based approaches	Meditation can be defined as *“a self-regulatory practice designed to “train attention in order to bring mental processes under greater voluntary control”* (Walsh, 1995, p. 388) (in Kenny, 2005, p. 195). Mindfulness- and acceptance-based approaches posit that the resistance to challenging emotions creates more problems than the emotions themselves, and therefore promotes awareness and acceptance of difficult experiences (Osborne and Kirsner, 2022) (For studies investigating Acceptance and Commitment Therapy and meditation/mindfulness approaches, please see Chang et al., 2003; Lin et al., 2008; Juncos et al., 2017; Juncos and de Paiva e Pona, 2018, 2022; Stanson, 2019; Clarke et al., 2020; Czajkowski et al., 2020; Shaw et al., 2020; Mahony et al., 2022).
Alexander technique	Alexander Technique is a kinesthetic education method which improves posture and body use through verbal instruction and challenges habitual contraction through intentionally directed inhibition or action. The method emphasizes economy of effort and managing tension to develop optimal physical functioning (Valentine et al., 1995). Only one formal study has investigated the therapeutic impact of Alexander Technique on MPA. In their study with 15 music students, Valentine et al. (1995) reported pre- to post-treatment decrease in MPA and increased positive attitude toward performance, but no changes were statistically significant. In addition to a lack of statistically significant results, Valentine et al. (1995) employed an inadequate sample size and insufficient data to calculate effect sizes for three out of five outcome measures (Kenny, 2005; McGrath, 2012). Furthermore, except HRV, effects were only visible in low stress situations. Overall, more robust research is required to clarify whether weak evidence for beneficial effects can be confirmed (Brugués, 2019; Fernholz et al., 2019).
Yoga	Yoga is a holistic system of practices incorporating cognitive and physical techniques including physical postures (designed to develop strength and flexibility) and breathing exercises (Khalsa et al., 2009). Results (Khalsa et al., 2009; Stern et al., 2012) indicate significantly decreased MPA, and significantly reduced generalized anxiety and depression, with benefits maintained at follow-up. Despite positive findings, all studies reviewed used small sample sizes and problematically, did not acknowledge the complexity of the intervention in terms of its combination of mind and body practices. Burin and de Lima Osório (2016) suggest that anxiety reduction could be attributed to meditation techniques and breathing training, rather than the physical aspect of yoga training. Further research is required to clarify which aspects of yoga training mediate which benefits, and to build a more robust evidence base (Fernholz et al., 2019).
Music therapy	Music Therapy is the clinical, evidence-based use of music-based interventions to achieve individualized outcomes within a therapeutic relationship by a qualified and accredited professional (https://www.musictherapy.org/) [See Cheng (2020), Montello (1989) and Montello et al. (1990) for studies investigating music therapy and MPA]. While results appear promising, more studies are needed with larger sample sizes (Brugués, 2019).
Biofeedback	Developed in the 1960s by experimental psychologist Neal Miller, biofeedback training is based on the theoretical premise that awareness is a necessary first step to changing one’s physiological state, and this awareness can enable new habits to be formed (Deen, 1999). Biofeedback training encompasses a range of techniques including heart rate variability (HRV) biofeedback, which involves slowing one’s breathing rate to regulate autonomic activity, and electromyographic (EMG) biofeedback, which measures a range of bodily functions including blood pressure, heart rate, muscular tension and skin temperature. In either type, participants’ physiological processes are fed back to them through sensors and real time on-screen monitoring, allowing them to gain control over these processes and thus alter their state. Additionally, biofeedback enables participants to identify the thoughts or emotions which trigger particular physiological responses (Niemann et al., 1993). In their study of 21 music students with severe MPA, Niemann et al. (1993) reported that biofeedback was effective in reducing MPA. However, the intervention’s complexity (combined biofeedback with CB strategies, e.g., coping, muscle relaxation, breathing awareness & imagery) precludes drawing any firm conclusions regarding biofeedback as a single treatment modality. Van McKinney (1984) (*n* = 32 wind players) found no effect on anxiety levels but reported improved performance quality (effect size = 0.83). However, Kenny (2005) attributes this improvement to increased familiarity with the performance situation during the study, questioning the validity of the claim. Thurber et al. (2010) investigated the impact of HRV biofeedback training and emotional self-regulation techniques on MPA in 14 university music students. Results indicated significant reductions in MPA and heart rate variability as well as improved performance quality. However, as the intervention combined HRV with mental and emotional refocusing strategies, it is unclear which component mediated the improvements. In their study of 46 musicians, Wells et al. (2012) found that HRV biofeedback training produced no differential results but breathing regulation significantly increased HRV and reduced self-reported anxiety. Overall, studies investigating biofeedback report mixed results. While a few studies report positive results (Niemann et al., 1993; Thurber et al., 2010), Van McKinney (1984) and Wells et al. (2012) found no reduction in MPA. Unfortunately, the studies all combine biofeedback with other treatment modalities, precluding any conclusions being drawn regarding its efficacy. McGinnis and Milling (2005) argue that although some biofeedback studies report positive change (Nagel et al., 1989; Niemann et al., 1993), it is impossible to differentiate the effects of biofeedback training from other components of the interventions. It may offer a beneficial option in conjunction with other modalities, but further research is required. Regarding biofeedback training alone, there is no good evidence to suggest it reduces MPA (Brugués, 2019). Studies investigating biofeedback training in isolation are needed to support its efficacy.
Hypnotherapy	The term ‘hypnosis’ refers to *“a state of physical relaxation accompanied and induced by mental concentration”*; in the context of interventions, the American Psychological Association defines hypnotherapy as a procedure used to “*encourage and evaluate responses to suggestions for changes in subjective experience, alterations in perception, sensation, emotion, thought, or behaviour”* (McGrath, 2012, p. 11). According to large-scale cross-sectional studies, hypnosis was rated as beneficial in managing MPA by 60–76% of musicians (Fishbein et al., 1988; D. Kenny et al., 2014; Middlestadt, 1990). However, very few studies have investigated empirically the impact of hypnosis on MPA (see Plott, 1986; Stanton, 1993). More recently, cognitive hypnotherapy has been combined with EMDR by Brooker (2018), showing promising results. Overall, there are insufficient data available to draw any conclusions regarding the efficacy of hypnosis for MPA (Brugués, 2019).
Other modalities	Oxytocin (Sabino et al., 2020) Expressive writing (Tang and Ryan, 2020) Depth relaxation music therapy and silence (Pfeifer et al., 2020)

#### 6.2.1. Methodological critique

From the studies cited in Table 5, one could reasonably infer that the state of play regarding MPA interventions is advanced, with a range of effective interventions to choose from. However, systematic reviews and meta-analyses tell a somewhat different story, reporting some positive evidence but arguing that evidence is largely weakened by methodological limitations. Although numerous MPA interventions have been developed and evaluated, it has not yet been possible to build a robust evidence base for any single intervention due to a range of methodological weaknesses within studies, methodological heterogeneity across studies and a lack of replication (McGinnis and Milling, 2005; Goren, 2014; Matei and Ginsborg, 2017; Fernholz et al., 2019; Zhukov, 2019).

One key methodological limitation is the lack of consistency in MPA definitions across studies and a lack of clarity regarding the theoretical and conceptual issues underpinning MPA within studies (Kenny, 2005). The terms MPA, performance anxiety, and stage fright continue to be used interchangeably across intervention studies, often without clarifying their exact meanings (Fernholz et al., 2019). The lack of a clear and consistent definition makes it difficult to know exactly what changes are occurring both within, and across, studies. Conceptual issues regarding MPA definitions will be discussed below. The lack of clarity regarding what constitutes MPA and the heterogeneity of MPA definitions used is, of course, reflected in the disparate outcome measures used, including some non-validated instruments (Kenny, 2005; Burin and de Lima Osório, 2016). In their systematic review, Fernholz et al. (2019) concluded it was often unclear exactly what was being measured, precluding clear interpretation of results within studies and comparison across studies and recommend using validated and standardized measurement tools.

As problematic as what is being measured, is what is not measured. A good evaluation is a crucial precursor to any intervention (Nagel, 2010), yet intervention studies often omit pre-test scores (Kenny, 2005; McGinnis and Milling, 2005; Zhukov, 2019). There is also a lack of process evaluations including adherence and acceptability, and of interrogation into specific components of interventions in terms of mediating and moderating variables (McGinnis and Milling, 2005), which is especially problematic in complex or combined interventions. A lack of follow-up data (longitudinal studies) makes it impossible to know long-term efficacy (Kenny, 2005; McGinnis and Milling, 2005).

Most studies use very small sample sizes, meaning that the statistical power is insufficient to justify definitive conclusions regarding interventions’ efficacy (Kenny, 2011; Goren, 2014; Zhukov, 2019). Overall, studies with larger and more homogenous subjects are needed (Brugués, 2011b). Studies often do not use a control group (Kenny, 2005; Matei and Ginsborg, 2017; Zhukov, 2019). This is especially problematic when studies use performance tasks to assess MPA, as differential results could be attributed to time spent practising, task familiarity or repeated exposure to the task. Few studies explicate the diagnostic inclusion/exclusion criteria used, or the level of MPA experienced by participants: heterogeneity in participants’ levels of MPA may obscure significant treatment effects (Kenny, 2011; Fernholz et al., 2019). In their systematic review, Burin and de Lima Osório (2016) reported that the majority of studies reviewed included no information regarding participants’ levels of MPA, whether normal or ‘pathological’ and argue that this information is crucial to draw conclusions regarding interventions’ efficacy in managing MPA. While it seems self-evident, McGinnis and Milling (2005) argue that research evaluating the efficacy of interventions designed to ameliorate MPA must be conducted with performers who actually experience MPA, and that findings based on participants with subclinical MPA levels cannot be used to support conclusions regarding interventions’ efficacy for MPA. Lastly, one reason it has not yet been possible to build a robust evidence base for any one intervention is the lack of standardized techniques utilized across different interventions within categories. The heterogeneity of treatment modalities, intervention duration and dosage (intensity) preclude comparison across studies of similar interventions (Kenny, 2005; Burin and de Lima Osório, 2016). Within individual studies, limitations include the absence of a manual detailing treatment protocol and the use of multiple therapists, or interventions being delivered by the principal investigator, risking researcher bias (McGinnis and Milling, 2005).

Based on their systematic reviews, Kenny (2005) and McGinnis and Milling (2005) concluded that there is an urgent need for more methodologically robust studies investigating MPA interventions, and highlighted key methodological weaknesses including conceptual ambiguities, inadequate assessment measures and small and heterogenous samples. Fifteen years later, the state of play remains much the same with recent systematic reviews recommending cautious interpretation of positive results based on a body of knowledge which remains inconclusive, inconsistent, and methodologically weak (Burin and de Lima Osório, 2016; Fernholz et al., 2019; Zhukov, 2019). In sum, key methodological limitations across intervention studies include disparate definitions and outcome measures, missing data, small and heterogenous samples, lack of control group, unclear inclusion/exclusion criteria and heterogenous intervention components within categories and lack of engagement with mechanisms of change.

If these methodological limitations of individual studies reporting positive change were the only issue, then replication would be the obvious next step. However, while these methodological limitations certainly weaken the evidence base for any one intervention, they do not explain why effects being reported at the study level are not seen at the population level. Poor-quality evidence does not mean interventions are not working, which poses an important question left unanswered by systematic reviews and meta-analyses: Why are interventions not having a meaningful impact on MPA? While there is no single answer, there are several possibilities worth exploring, including accessibility issues, MPA conceptualizations, and excessive focus on symptoms.

#### 6.2.2. Conceptual critique

Based on individual studies, it is entirely plausible that effective interventions *do* exist, but that their impact is limited due to *accessibility barriers* including prohibitive cost or ineffective dissemination. For example, Spahn et al.’s (2016) multimodal intervention seems highly effective, but its individually tailored design precludes widespread availability, and it has not yet entered the mainstream of interventions – it is unclear where or how one would access it. Overall, empirically-validated cost-effective and accessible interventions are lacking.

In addition to logistical accessibility issues, musicians may not seek help due to the *stigma* (both real and perceived) still associated with MPA (McGrath, 2012; van Fenema et al., 2013). While there is now seemingly greater openness around physical injuries (Horvath, 2001; Brandfonbrener, 2004), research suggests that this openness has not yet reached psychological issues, with musicians reportedly associating MPA with personal weakness and/or shame (Juncos et al., 2017). As stigma (associated with feelings of shame, disgrace or secrecy) plays a significant role in determining health-seeking behaviors outside of music (Eisenberg et al., 2009; Bharadwaj et al., 2017), this could logically apply to musicians as well.

As discussed in Section 3.2.1, MPA is predominantly conceptualized pathologically, which implies that it is an affliction to be prevented, avoided or eliminated to perform well and happily (Senyshyn and O’Neill, 2001). Based on this *conceptualization*, interventions largely seek to manage MPA through ameliorating symptoms. Although logical, this approach is problematic in a number of ways. Firstly, there is an implicit assumption that fewer or no ‘symptoms’ is the desired outcome, yet research consistently indicates that some anxiety (or arousal) is intrinsic, even beneficial, for performance (Kenny, 2005; Matei and Ginsborg, 2017) and that reducing anxiety may not be a useful, or even optimal, goal (Kirchner, 2003; Nagel, 2010; Lawrence, 2019). Indeed, relaxation is both unrealistic and counterproductive for peak performance in high-pressure contexts (Pecen et al., 2016), as illustrated by these quotes:

*“Relax at the Tchaikovsky competition? Relax when you’ve blown $900 to fly across the country for your two-minute audition for the only symphony flute job to come open this year? Relax when your symphony job security depends on near-perfect technique in each and every concert until you’re tenured? I don’t think so”* (Greene, 2012, p. 1).

*“Trying to calm down before a stressful performance may not only be futile – but counterproductive to boot!”* (Kageyama, 2022).

Secondly, research suggests that ‘symptoms’ themselves may in fact not be the issue. In their evaluation of a mental skills training program for conservatoire students, Clark and Williamon (2011) found no significant differences in state or trait anxiety post intervention, yet participants reported more facilitative views toward MPA, indicating altering one’s interpretation may alleviate MPA’s impact more effectively than reducing it. While MPA research has largely focused on whether performers interpret MPA as either positive or negative, research outside of the musical domain suggests that anxiety-related psychopathology is characterized by experiential *avoidance* of fear, not the *experiencing* of it. In this framework of understanding anxiety, the reaction *to*, not the presence *of,* anxiety is where problems arise, which has important implications for how we define MPA – perhaps not as the anxiety itself, but in the reaction to the anxiety?

Thirdly, focusing on the *presence* of ‘symptoms’ does not address the underlying mechanisms by which MPA can disturb performance, as discussed in Section 5.3. Focusing on these mechanisms (e.g., interaction, interpretation and attention regulation) may be more effective.

Fourthly, excessive focus on symptoms overlooks the constellation of other factors associated with MPA, including personality traits and occupational stress, as discussed in Section 4. Indeed, several interventions focus only on one domain (cognitions or behaviors), thus not sufficiently addressing MPA’s multidimensionality (Brandfonbrener, 1999). Interventions which neglect underlying psychological factors are unlikely to succeed (Pecen et al., 2016). Conceptualizing MPA as a multidimensional construct involving complex interaction between the individual and their environment suggests a more holistic approach to its management: MPA interventions should ideally address the full range of MPA processes alongside the numerous vulnerability factors associated with it (Osborne and Franklin, 2002).

Lastly, efforts to reduce MPA largely rely on intentionally changing one’s state (e.g., from anxious to relaxed). There is growing awareness that efforts to reduce unwanted experience creates resistance, inadvertently exacerbating anxiety, as illustrated by this quote:

*“Any attempt to not feel the fear splits the performer psychically into two persons, the feeler and the repressor. It is the splitting, not the fear, that limits capability…it is the attempt not to feel rather than the feeling that impairs the performance”* (Conable and Conable, 1995, p. 115).

Despite the wide range of interventions on offer, there remains no established way of dealing with MPA. Section 7 will briefly summarize the current state of play, before suggesting a reconceptualization of MPA.

## 7. Discussion: summary and reconceptualization

MPA affects numerous musicians across all ages, nationalities and musical genres. It occurs in response to threat and manifests in physiological, cognitive, emotional and behavioral ‘symptoms’ which can negatively affect performer and/or performance. It co-varies with a wide range of maladaptive personality traits as well as situational stressors associated with the performance context and is clustered diagnostically within the anxiety disorders landscape. MPA is widely considered to be a significant occupational hazard, and musicians use a range of coping strategies to manage it, many of which are ineffective and/or unhealthy. Despite the existence of numerous interventions, unchanged prevalence figures indicate these are not having a meaningful impact. After decades of research, we still do not really know how best to offer appropriate support to musicians experiencing MPA. We suggest that prevailing views on MPA are conceptually flawed and crucially, not serving musicians.

Zooming out briefly, the broader conversation around mental health has seen a shift over the last 40 years, from the biomedical model traditionally used in psychiatry, to the biopsychosocial model (Engel, 1977). This model emphasizes the importance of psychological and social factors alongside biological factors in understanding illness (Alonso, 2004). Although still awaiting empirical support, Manchester (2011) and Mumm et al. (2020) have convincingly proposed applying the biopsychosocial model to understanding MPA’s etiology. While the biopsychosocial model offers a useful heuristic to understand the many factors associated with MPA’s development, we suggest moving away from both the biomedical and biopsychosocial models, as both are used to describe illness. We propose removing any medical connotation from MPA and reconceptualizing it as an adaptive response to a unique constellation of demands and stressors, which will now be discussed.

### 7.1. The soil MPA grows in

As discussed in Section 4.2, MPA is associated with/predicted by a number of *intrinsic factors* including trait anxiety, neuroticism, low self-esteem, maladaptive dimensions of perfectionism, guilt- and shame-proneness, and maladaptive cognitive style characterized by rumination, fear of negative evaluation and judgmental attitude. Across relevant literature, these traits are (implicitly) viewed as individual deficiencies, with minimal discussion of the soil they grow in.

Research clearly shows the challenges facing musicians on the path to a professional career, including fear experienced due to excessive demands from parents or teachers (Mumm et al., 2020), managing the consequences of early specialization, grueling practice schedules, social isolation, psychological pressure (Pecen et al., 2016), neuromuscular and musculoskeletal overuse and irregular sleep and work schedules (Pecen et al., 2018). In her book ‘Music from the Inside Out’, Tomlinson (2018) writes

*“In our culture, judgement underpins everything. Learning to play an instrument to professional standards is unbelievably demanding. You need skill, commitment and discipline; you need to master your instrument, faithfully interpret the music you are playing, manage your nerves and above all, be free enough to express yourself. And if you are doing this in a culture that has judgement at its core, there are consequences: judgement is dangerous”* (p. 3).

If we look at MPA as a societal rather than an individual phenomenon, many, if not all, of the traits associated with MPA start to seem less like coincidental, intrinsic deficiencies, and more like natural, even predictable, adaptations to years of intensive musical training in competitive and often un-nurturing environments that have judgment at their core, as Tomlinson suggests. Arguably, to be a classical musician and NOT a perfectionist, or neurotic, or fear negative evaluation, would be a radical act of exceptionalism given the educational and professional cultures within which musicians often spend their formative years. We turn now to the *extrinsic factors* associated with MPA’s etiology.

### 7.2. Performance demands

Research unequivocally highlights the demands of musical performance, including the relentless pressure to perform to near-perfect levels (McNeil et al., 2022), executing complex motor, cognitive, psychological and emotional skills/processes under close scrutiny and in highly anxiogenic situations (Hays, 2009; Antonini Philippe et al., 2019; de Figueiredo Rocha, 2020). The success and longevity of a musical career depends on musicians’ ability to delivery consistently high-quality performances under pressure (Kenny, 2011; Williamon et al., 2013). While musicians are often compared to athletes in terms of the psychophysical demands of elite performance, musicians receive none of the specialist training or support routinely offered to athletes, including physiotherapy, nutrition, and psychological skills training.

Compounding the pressures of performance are the realities of working within an insecure and highly competitive profession – well-documented occupational challenges include long and anti-social working hours, adverse working conditions, the challenges of touring and financial unpredictability (Voltmer et al., 2012; van Fenema et al., 2013; Sousa et al., 2016). Based on their 2016 study investigating musicians’ mental health, Gross & Musgrave write *“Music making is therapeutic but making a career out of music is destructive…whereas artists find solace in the production of music, the working conditions of forging a musical career are traumatic.”* (2016, p. 12).

Unsurprisingly, sustaining the demands of high-level performance against the backdrop of a challenging profession often generates stress and anxiety for performers (Antonini Philippe et al., 2022). Indeed, research suggests that managing this anxiety is a *“constant battle even for the most accomplished musicians and performers”* (Tang and Ryan, 2020, p. 1). Seemingly, MPA poses a challenge that many, if not all, performers must meet (McGinnis and Milling, 2005; Spahn et al., 2010), suggesting that it is just part of the fabric of life as a performing musician. As Hays (2009) asks, “*when a condition is ubiquitous, is it diagnosable?*” (p. 105), succinctly problematizing diagnosing a ‘normative aspect of performance’.

### 7.3. Conclusion

In 2020, biological anthropologists Syme and Hagen (2020) published a ground-breaking paper in which they suggest reclassifying ‘diseases of the mind’ in an entirely new framework consisting of: (1) highly heritable (rare) disorders including schizophrenia and OCD, (2) disorders associated with age-related deterioration such as Alzheimer’s disease, (3) a mismatch between ideal and modern environments including ADHD and (4) functional responses to adversity, including PTSD and anxiety disorders. We propose a similarly radical paradigm shift – removing any medical connotation from MPA and reconceptualizing it as a *functional response to adversity*, where adversity represents the intersection between managing the demands of high-pressure performing situations, a competitive and insecure professional environment, and personality traits associated with elite musical training. Acknowledging this wider context seems key in understanding how MPA develops and sustains, not as an individual deficiency, but as a functional response to a highly challenging education and performance environment.

The World Health Organization (WHO, 2004) defines anxiety as a “normal and healthy reaction to perceived danger that triggers a variety of physical, mental, and behavioral changes in order to facilitate a speedy response” (cited in Osborne and Kirsner, 2022, p. 206). While performing a Mozart concerto may not be the same as encountering a hungry tiger, we suggest there are many rational reasons why performers might, on some level, perceive performing as threatening. The vulnerability and exposure inherent in performance, as well as the reliance on the positive opinion of others (colleagues, critics, agents, fixers, concert promoters or audiences) to pay one’s rent, arguably pose a very real and practical threat, making anxiety a rational response. The recent headline news regarding Arts Council England and the BBC funding cuts offer a flavor of the fragility of the musical ecosystem and the very real threat this poses to musicians’ livelihoods and wellbeing.

Anxiety is a biologically adaptive response, which has ensured human survival for millennia. Although MPA is almost universally described using medical language (‘*symptoms’, ‘disorder’, ‘treatment’* etc.), we suggest that the body’s ability to react to what it perceives as threatening is not a sign of illness, but rather signifies a functioning autonomic nervous system. As an analogy: smoke alarms exist to detect and alert us to fire. If our smoke alarm activates in response to burnt toast, we would not disable it, but rather understand that an overly-cautious alarm is preferable to the opposite. We might open a window or wave a tea towel at the agitated alarm, to reassure it that there is no fire. Imagine if we could similarly learn to reassure our nervous system – to understand that it is doing exactly what it evolved to do, and to thank it for its tireless service, to see it as a protector, not a disease.

We question the use of medicalized language and propose using more neutral terms ‘response’, ‘manifestation’, or ‘experience’. This may seem like semantics, but consider the felt impact on a performer when discussing their ‘symptoms’ – it immediately conjures up images of infection or disease – MPA is arguably neither, and we suggest that using the same language to describe it is deeply problematic. Additionally, diagnostic labeling has ‘*pejorative implications for normative behaviour’* (Hays, 2009) and arguably perpetuates the stigma, shame and silence still surrounding MPA (Patson and Loughlan, 2014). Re-imagining ‘symptoms’ as ‘the body seeking to protect itself from threat, as organisms evolved to do’ could open up new ways of thinking about how we meet challenging experiences. In sum, MPA is not a virus! Reconceptualizing MPA as a normal and adaptive response to the well-documented stressors associated with performance and a competitive and insecure profession, could profoundly change the conversation around how performers can be supported throughout their musical lives.

## 8. Potential developments: implications for theory and practice

Our proposed reconceptualization of MPA, from disorder to adaptive response, has implications for both theory and practice, which will now be discussed.

### 8.1. Implications for theory

Based on this review, we suggest the following as areas for potential future research:

- Terminological clarity: Using the same term (‘MPA’) to describe a phenomenon which can be positive, mildly challenging, a psychological disorder or a completely normal part of a performer’s life seems at best, unhelpful, at worst deeply problematic. We suggest that developing a coherent MPA theory requires greater terminological clarity.
- Conceptual clarity: A new MPA definition which moves away from the medical model and encompasses the complexity and individuality with which MPA manifests, while acknowledging the adaptive role MPA plays in terms of preparing the body to deal with perceived threat.
- A new standardized assessment tool is required to reflect a broader conceptualization of MPA, and to enable comparability across intervention studies from different theoretical perspectives.
- As research has largely focused on debilitating MPA, those who experience MPA positively and/or manage it successfully are underrepresented in scientific literature, highlighting an important gap in current understanding.
- Impact of MPA on performer and performance: the factors which mediate the intensity and interpretation of MPA (and thus its impact on performer and/or performance) are currently scattered across the literature. Further research is needed to amalgamate these factors into one coherent theory which accounts for MPA’s impact on performance and/or performer.
- MPA Etiology – Longitudinal research investigating the development of traits which predict or protect against MPA within musical training environments such as specialist music schools and conservatoires, as well as qualitative research to better understand the relationship between MPA and its comorbidities.

### 8.2. Implications for practice: the role of music education

Given the importance of coping with MPA in establishing and maintaining a successful performing career, you might think that it would be core business for music schools and conservatoires. Indeed, a few institutions have integrated empirically evaluated interventions into their curricula – see Candia et al. (2023) and Spahn et al. (2016) for notable examples. However, this is far from standard practice – research shows that teaching effective coping is not yet embedded in musical training (Pecen et al., 2016; Araújo et al., 2017) [As a comprehensive review of the field of music education is beyond the scope of this paper, please see the following sources for further discussion: Hildebrandt (2009), Hildebrandt and Nübling (2004), and Mazzarolo et al. (2023)].

We suggest that music education plays a critical role in the formation of perceptions and beliefs around performance, and is therefore a formative place for the development of a problematic or constructive approach to MPA. Where else than in music education – from the earliest music lessons to the training of professionals and future teachers – can a reconceptualization of MPA and the integration of effective coping strategies succeed? We suggest that musical training should, as standard practice, (1) include psychoeducation regarding the physical and psychological demands of performance (including understanding the body’s inbuilt stress response), and (2) the training of effective and empirically-validated practice and performance strategies to manage these demands [see Hildebrandt (2009) for an example of a learnable ‘stage disposition’ which includes the training of performance-appropriate distribution of muscle tension and attentional focus for peak performance]. Equipping students with a solid understanding of MPA as well as the skills to manage it seems vital in preparing the next generation of musicians to perform healthily and happily.

## Author contributions

RH conducted the literature search for this review and wrote the first draft of the manuscript. RH and TC developed the ideas for both content and structure. Both authors contributed to manuscript revision, read, and approved the submitted version.
