# Corrigendum: It's not a virus! Reconceptualizing and de-pathologizing music performance anxiety

**DOI:** 10.3389/fpsyg.2025.1590188

**Published:** 2025-06-02

**Authors:** Rebecca Herman, Terry Clark

**Affiliations:** ^1^Centre for Performance Science, Royal College of Music, London, United Kingdom; ^2^Mount Royal Conservatory, Calgary, AB, Canada

**Keywords:** music performance anxiety, MPA, musicians, interventions, theoretical literature review

In the published article, there was an error in quoting Dianna Kenny's definition of MPA, the citation provided, and the subsequent critique. The correct definition, citation, and revised critique are offered below.

A correction has been made to section *3.3. Kenny's definition, Paragraphs 1-4*.

The text previously stated:

## 3.3 Kenny's definition

The definition most widely used today is offered by Kenny (2009):

“*The experience of marked and persistent anxious apprehension related to musical performance that has arisen through specific anxiety-conditioning experiences and which is manifested through combinations of affective, cognitive, somatic, and behavioural symptoms. It affects musicians for their entire lives and is at least partially independent of years of training, practice, and level of musical accomplishment. It may or may not impair the quality of the musical performance”* (2009, p. 433).

Although this definition is used by most contemporary MPA studies, there are issues worth discussing. Firstly, defining MPA by its ‘*symptoms'* perpetuates the pathologizing narrative, conjuring up images of illness and disease. As well as the philosophical issues with medicalizing MPA (see Section 7), if it can be facilitative for some, or simply inherent to performance, then the *presence* of ‘symptoms' may not be the key issue to understanding or managing MPA.

Secondly, there is no empirical support for the assertion that MPA “*affects musicians for their entire lives.”* Are there really no musicians who have managed to overcome it? This argument presumably stems from the issue that MPA studies are pathologically focused, with little account across literature of musicians who successfully manage, or indeed overcome, MPA. Indeed, many studies are fairly cross-sectional, or at least of a limited duration, as opposed to truly long-term, meaning there are minimal (if any) longitudinal data investigating MPA management. Thirdly, MPA is “*at least partially independent of years of training, practice, and level of musical accomplishment.”* Given that MPA is reported by individuals of all levels of training, experience and expertise (including celebrated artists such as Chopin, Casals, Rubinstein, Horowitz and Rachmaninoff), MPA can presumably be *entirely*, not *partially*, independent of expertise (Brugués, 2011a; Kantor-Martynuska et al., 2018).

Lastly, the strand “*MPA may or may not impair the quality of the musical performance”* tells us very little about the complex relationship between MPA and performance quality, which will be discussed in Section 5.3. It also omits the impact MPA can have on the *experience* of performing, regardless of whether quality is affected. Across the extensive landscape of MPA literature, a recurring theme is the significant variability with which MPA can manifest, ranging from performance-enhancing, to minimally negative, to debilitating, to career-ending and varying in terms of regularity, performance-setting and manifestation (Nagel et al., 1989; Van Kemenade et al., 1995; Miller and Chesky, 2004; Fehm and Schmidt, 2006; Patson and Loughlan, 2014; Lawrence, 2019). This complexity and multidimensionality is arguably not yet reflected in the prevailing approach to defining MPA.

The corrected text appears below.

The definition most widely used today is offered by Kenny (2010):

“*Music performance anxiety is the experience of marked and persistent anxious apprehension related to musical performance that has arisen through specific anxiety-conditioning experiences. It is manifested through combinations of affective, cognitive, somatic and behavioural symptoms and may occur in a range of performance settings, but is usually more severe in settings involving high ego investment and evaluative threat. It may be focal (i.e., focused only on music performance), or occur comorbidly with other anxiety disorders, in particular social phobia. It affects musicians across the lifespan and is at least partially independent of years of training, practice, and level of musical accomplishment. It may or may not impair the quality of the musical performance.”* (p. 433)

Although this definition is used by most contemporary MPA studies, there are issues worth discussing. Firstly, defining MPA by its “*symptoms”* perpetuates the pathologizing narrative, conjuring up images of illness and disease. As well as the philosophical issues with medicalizing MPA (see Section 7), if it can be facilitative for some, or simply inherent to performance, then the *presence* of “symptoms” may not be the key issue to understanding or managing MPA.

Secondly, MPA is “*at least partially independent of years of training, practice, and level of musical accomplishment.”* Given that MPA is reported by individuals of all levels of training, experience and expertise (including celebrated artists such as Chopin, Casals, Rubinstein, Horowitz, and Rachmaninoff), MPA can presumably be *entirely*, not *partially*, independent of expertise (Brugués, 2011a; Kantor-Martynuska et al., 2018).

Lastly, the strand “*MPA may or may not impair the quality of the musical performance”* tells us very little about the complex relationship between MPA and performance quality, which will be discussed in Section 5.3. It also omits the impact MPA can have on the *experience* of performing, regardless of whether quality is affected. Across the extensive landscape of MPA literature, a recurring theme is the significant variability with which MPA can manifest, ranging from performance-enhancing, to minimally negative, to debilitating, to career-ending and varying in terms of regularity, performance-setting and manifestation (Nagel et al., 1989; Van Kemenade et al., 1995; Miller and Chesky, 2004; Fehm and Schmidt, 2006; Patson and Loughlan, 2014; Lawrence, 2019). This complexity and multidimensionality is arguably not yet reflected in the prevailing approach to defining MPA.

The authors apologize for this error and state that this does not change the scientific conclusions of the article in any way. The original article has been updated.
